# Genetic Predisposition to the Mortality in Septic Shock Patients: From GWAS to the Identification of a Regulatory Variant Modulating the Activity of a *CISH* Enhancer

**DOI:** 10.3390/ijms22115852

**Published:** 2021-05-29

**Authors:** Florian Rosier, Audrey Brisebarre, Claire Dupuis, Sabrina Baaklini, Denis Puthier, Christine Brun, Lydie C. Pradel, Pascal Rihet, Didier Payen

**Affiliations:** 1 Aix Marseille Univ, INSERM, TAGC, UMR_S_1090, MarMaRa Institute, 13288 Marseille, France; florian.rosier@etu.univ-amu.fr (F.R.); a.brisebarre@laposte.net (A.B.); sabrinabaaklini@gmail.com (S.B.); denis.puthier@univ-amu.fr (D.P.); christine-g.brun@inserm.fr (C.B.); 2 Medical Intensive Care Unit, Clermont-Ferrand University Hospital, 58 rue Montalembert, 63003 Clermont-Ferrand, France; cdupuis1@chu-clermontferrand.fr; 3 CNRS, 13288 Marseille, France; 4 UMR INSERM 1160: Alloimmunité, Autoimmunité, Transplantation, University of Paris 7 Denis Diderot, 2 rue Ambroise-Paré, CEDEX 10, 75475 Paris, France

**Keywords:** sepsis, GWAS, SNPs, non coding region, *CISH*, enhancer, CRISPR-Cas9, luciferase assay

## Abstract

The high mortality rate in septic shock patients is likely due to environmental and genetic factors, which influence the host response to infection. Two genome-wide association studies (GWAS) on 832 septic shock patients were performed. We used integrative bioinformatic approaches to annotate and prioritize the sepsis-associated single nucleotide polymorphisms (SNPs). An association of 139 SNPs with death based on a false discovery rate of 5% was detected. The most significant SNPs were within the *CISH* gene involved in cytokine regulation. Among the 139 SNPs associated with death and the 1311 SNPs in strong linkage disequilibrium with them, we investigated 1439 SNPs within non-coding regions to identify regulatory variants. The highest integrative weighted score (IW-score) was obtained for rs143356980, indicating that this SNP is a robust regulatory candidate. The rs143356980 region is located in a non-coding region close to the *CISH* gene. A CRISPR-Cas9-mediated deletion of this region and specific luciferase assays in K562 cells showed that rs143356980 modulates the enhancer activity in K562 cells. These analyses allowed us to identify several genes associated with death in patients with septic shock. They suggest that genetic variations in key genes, such as *CISH*, perturb relevant pathways, increasing the risk of death in sepsis patients.

## 1. Introduction

The incidence of sepsis, particularly severe sepsis and septic shock, is increasing among hospital transfer, with a mortality rate between 20 and 35% despite improved strategies of care [1]. After the analysis of negative results from randomized clinical trials (RCTs) to reduce mortality after 28 days and/or 90 days [2,3,4,5], new aspects of sepsis have come to the front. The demonstration of systemic and tissue immuno-depression after a septic injury [6] and the impact of co-morbidities [7] both motivate a change in the sepsis syndrome paradigm [8]. Research on the genetic predispositions associated with outcomes and polymorphisms for genes encoding mediators of inflammation, such as TNFα [9] and IL-10 [10], has been poorly replicated [11], and negative results on crude mortality reduction were obtained when agents blocking TNFα were tested. Despite the large number of such studies, re-analyses of accumulated evidence do not definitely show any associated genes [11]. As many other complex syndromes for which environmental and chronic disease risk factors are thought to interact with multiple genes, such analyses may benefit from recent genetic methodologies, such as genome-wide association studies (GWAS) [12]. Instead of sepsis syndrome, the GWAS approach has provided important genetic and biological insight for other more specific infectious diseases, such as meningococcemia or malaria [13,14].

An article by Rautanen et al. [15] presents the results from the first GWAS on survival from sepsis due to pneumonia, which was assessed in a multistage study, including four cohorts and tested almost 6 million single nucleotide polymorphisms. The authors identified genetic variants within the *FER* gene showing consistent effects across the four cohorts studied. The rs4957796 C allele, with a near 20% frequency in European populations, was associated with reduced mortality in sepsis caused by pneumonia. Scherag et al. conducted another GWAS in patients with severe sepsis. They reported 14 loci with suggestive evidence of an association with 28-day mortality [16]. Nevertheless, they did not find an association between rs4957796 and 28-day mortality and no evidence on an association between other loci identified by Rautanen et al. and 28-day mortality. They proposed that the focus on pneumonia-induced sepsis by Rautanen et al. may explain the observed discrepancies. In the same way, it was proposed that genetic studies should focus on specific traits related to severity and outcomes rather than on a broadly defined syndrome [17].

We have collaborated to provide the first human GWAS on severe sepsis or septic shock using the randomized control trial PROWESS database [18] to test the benefits of activated protein C (aPC) use on outcome. Because one arm of the trial received aPC treatment, the sepsis prognosis models have only been tested on the control arm. The prognosis was finally dominated by clinical variables with modest relation with the tested genetic markers. The second randomized control trial PROWESS SHOCK [3] tested aPC exclusively on septic shock patients and failed to show a crude outcome benefit at day 28 or 90. This negative result of aPC treatment in septic shock prompted us to perform GWAS on outcome specific traits using the complete PROWESS dataset after selection of septic shock patients. The present study reports the first identification of genetic variants associated with the prognosis of septic shock when comorbidity levels and systemic inflammation intensities are integrated. Since the mechanisms for early death differ from those causing late death [19,20], we also investigated the association of genetic variants with both early and late death. We further focused on a non-coding region significantly associated with late death and showed its regulatory activity on one of the closest gene, the cytokine inducible SH2 containing protein *(CISH)* gene. Interestingly, CISH is known to be a negative regulator of cytokine signaling.

## 2. Results

### 2.1. Patients and Covariates

Tables S1 and S2 summarize the clinical characteristics and the differences between groups with early or late deaths. The patients who died early were older, had higher IL-6 plasma levels at day 1 than other patients. Patients who died after day 7 were also older than the survivors and had a higher incidence of cardiomyopathy. For the genetic association studies, only the significant parameters were included in the model. For the early death analysis, the data were adjusted for age, IL-6 level, and sequential organ failure assessment (SOFA) score. For the late death analysis, the data were adjusted for age, cardiomyopathy presence, aPC treatment, and SOFA score.

### 2.2. Genome-Wide Association Analysis

Tables S1 and S2 list the single nucleotide polymorphisms (SNPs) considered significant based on a false discovery rate (FDR) of 5%. On this basis, we identified 32 SNPs and 107 SNPs associated with early and late death, respectively. Table 1 lists the SNPs selected after the Bonferroni correction. Here, we describe the genes and their genetic variants using the following criteria: (1) the gene contains at least one SNP associated with mortality based on a threshold of significance of approximately 5.58 × 10^−8^ when the Bonferroni correction was used; (2) the SNP is located within a gene selected on the former criteria, and is associated with mortality with an FDR of 5%; (3) the gene, for which we anticipate a functional relevance in sepsis.

**Figure 1:**
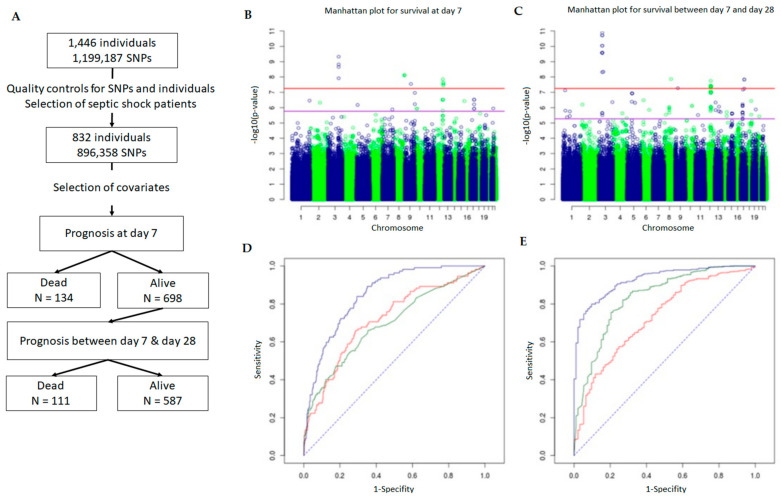
SNPs selection design (**A**). Schematic outline of patient and SNP selection. After quality control of the SNP data, 896,358 SNPs and 832 patients having septic shock were selected for genome-wide association analysis. (**B**–**E**) Genome-wide association results for early and late mortality. Manhattan plots show the −log10 (*p* value) for the association of SNPs with early (**B**) and late (**C**) mortality according to their position on the genome. The horizontal red line and the purple line correspond to a Bonferroni threshold and an FDR of 5%, respectively. Receiving operating characteristic (ROC) curves plotting sensitivity against 1-specificity are shown for the prediction of early (**D**) and late (**E**) mortality based on the effect of covariates/confounding factors (red), SNPs (green), and both (blue).

Early mortality: the GWAS analysis identified 12 SNPs after Bonferroni correction (Figure 1B, Table 1 and Table S1). For the identified SNPs, the minor allele was associated with a higher risk of mortality (Table 1). These 12 SNPs correspond to 4 loci on chromosomes 3, 8, 9, and 12. The three intragenic SNPs having the strongest evidence of association with early death (Table 1) were located within *CYP11B2*: rs11991278; rs6981918, and rs4544. A strong association was also observed between early death and rs7974468 located within *PTPN11* and rs956727 located in *SLC28A3*. Moreover, four other SNPs located within *PTPN11* (rs11066321, rs9668774, rs7975439, and rs12301915) were associated with mortality based on an FDR of 5% (Table S1).

Mortality between days 7 and 28: The GWAS analysis identified 16 SNPs after Bonferroni correction (Figure 1C, Table 1 and Table S2). The three intragenic SNPs having the strongest evidence of association with late death (Table 1) were located within *CISH* (rs2239753), *CACNA2D2* (rs12491812), and *MAPKAPK3* (rs12492982). Two other SNPs within *CISH* were also strongly associated (rs2239751 and rs2239752) (Table 1), as was rs7953683 located in *PAWR*. In addition, six other SNPs close to *CISH* and located within *DOCK3* or *MAPKAPK3* were associated (Table 1). Moreover, 17 additional SNPs within *PAWR* and 1 SNP within *CACNA2D2* (rs1107312) were associated with mortality based on an FDR of 5% (Table S2).

To summarize, we identified 139 SNPs associated with mortality in patients with septic shock. Noticeably, the SNPs associated with early death are different from those associated with late mortality. This supports the hypothesis that molecular mechanisms causing early death are at least partly different from those causing late death. Besides, the intragenic SNPs with the most significant *p* values for early death were within *CYP11B2*, which is involved in the renin-angiotensin-aldosterone system. Interestingly, the SNPs displaying the best *p* values were within the *CISH-MAPKAPK3-DOCK3* locus. The *CISH* and *MAPKAPK3* genes are known to modulate the immune response [21,22,23,24,25,26], whereas *DOCK3* is mainly expressed in nervous system and involved in developmental disorders [27,28,29].

### 2.3. Usefulness of GWAS to Predict Septic Shock Outcome

A genetic score was calculated based on the leading SNP at each of the 15 loci among the SNPs that were associated with early mortality after setting up an FDR of 5% (see the Table S1). The leader SNPs were rs16857836, rs11991278, rs7974468, rs10849641, rs956727, rs11137198, rs9891869, rs16840396, rs2061815, rs34737153, rs11948550, rs2838103, rs16928895, rs12268257, and rs17169594. The analysis showed that individuals with 4 or more risk alleles had a death risk 12.33-fold higher than those with no risk alleles (adjusted OR = 12.33, 95% CI 5.19–31.82) (Table 2). The cumulative effect of the risk alleles is also represented by receiving operating characteristic (ROC) curves (Figure 1D). The addition of SNPs significantly improved the AUC obtained with the clinical covariates (0.72 to 0.85; *p* = 3.15 × 10^−13^).

In the same way, a genetic score was calculated based on the leading SNP at each of the 32 loci among the SNPs that were associated with late mortality after setting up an FDR of 5% (see the Table S2). For survival between day 7 and day 28, the leader SNPs were rs359952, rs17442970, rs6692946, rs1509380, rs2239753, rs17072628, rs9856368, rs6852672, rs12654328, rs7726677, rs3797817, rs6910170, rs11987625, rs11994554, rs7840669, rs3005838, rs7096890, rs4575240, rs7953683, rs1882182, rs527603, rs7992136, rs4646220, rs1756650, rs7178141, rs2340518, rs1434590, rs7214197, rs1502522, rs4381690, rs17271418, and rs2232619. The results show that individuals with four or more risk alleles have a death risk 123.35-fold higher than those with no risk alleles (adjusted OR = 123.35, 95% CI 23.64–2292) (Table 2). The cumulative effect of the risk alleles is also represented by ROC curves (Figure 1E). The addition of SNPs significantly improved the AUC obtained with clinical covariates (0.73 to 0.93; *p* = 9.69 × 10^−23^).

In conclusion, our results provide evidence of a strong cumulative effect of genetic factors on both early and late mortality.

### 2.4. Protein–Protein Networks and Functional Enrichments

**Figure 2:**
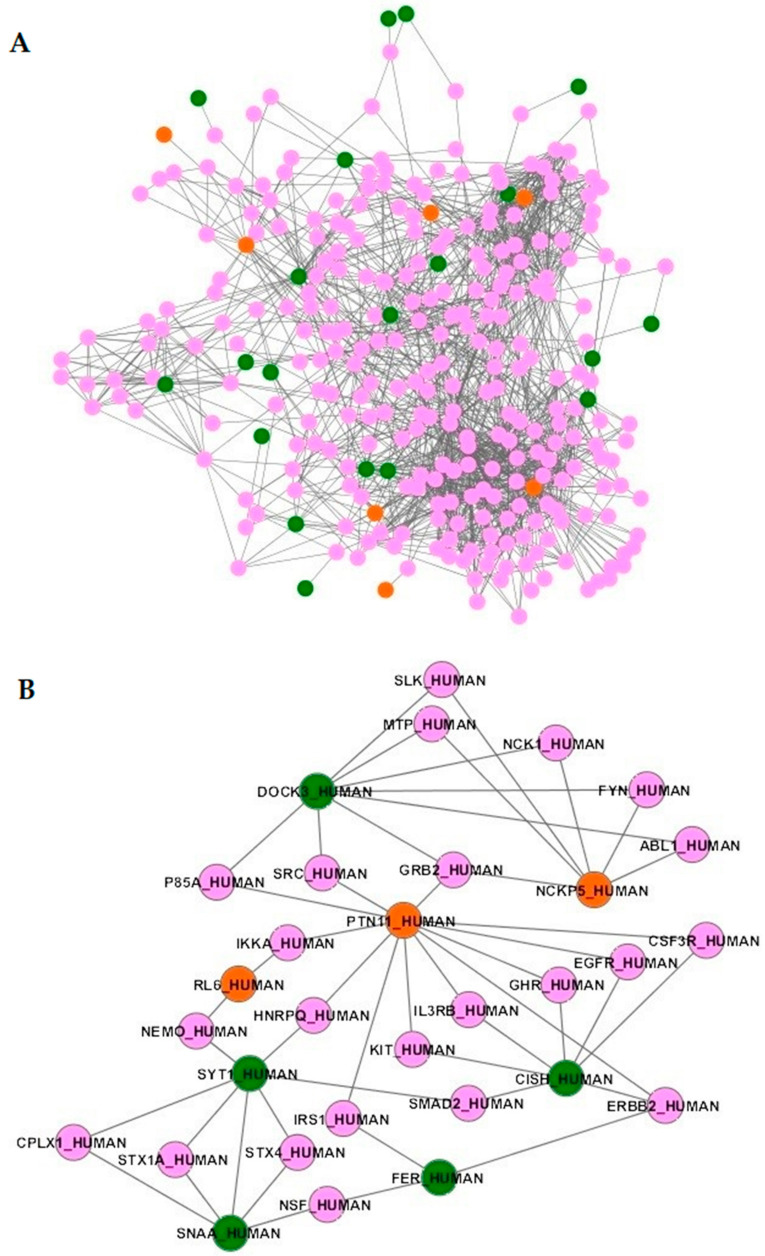
Subnetwork of the 27 connected proteins associated with sepsis and their direct interactors. Orange proteins are those encoded by genes associated with early mortality. Green proteins are those encoded by genes associated with late mortality. Pink proteins are direct interactors of proteins encoded by associated genes. The global network (**A**) and zooms on proteins of interest (**B**) are shown.

Using the 45 genes containing SNPs associated with early or late mortality or in linkage disequilibrium with those SNPs (Table 3), no functional enrichment was detected (data not shown). The products of those genes were further mapped onto a high-quality human protein network [30]. Only the protein products of 30 genes were mapped, as 15 genes encoded proteins were not present in the network (Table 3). Interestingly, 27 out of these 30 proteins encoded by genes associated with mortality are particularly close in the network. Indeed, average characteristic path lengths for the sepsis network and for the whole protein network were 3.16 and 3.79, respectively. The distribution of characteristic path length of the sepsis network significantly differed from that of the whole protein network (*p* < 0.0001). We therefore extracted the subnetwork containing 1617 interactions between 325 proteins (Figure 2A and Table S3), including all the interactors of the 27 proteins. As an example, Figure 2B shows the proteins interacting with PTPN11, CISH, FER, NKCP5, DOCK3, RL6, SYT1, and SNAA/NAPA, the SNPs of which have been associated with early or late death. Furthermore, this subnetwork was significantly enriched for 79 biological pathways with *p*-values corrected for multiple tests lower than 0.05 (Table S4). These pathways included those related to the renin-angiotensin-aldosterone system (aldosterone-regulated sodium reabsorption) and many pathways related to the immune system, which can be clustered into immune signaling pathways (“MAPK signaling pathway”, “T cell receptor signaling pathway”, “Toll-like receptor signaling pathway”, “Natural killer cell mediated cytotoxicity”, “NF-kB signaling pathway”, “Jak STAT signaling pathway” and “IL6 signaling pathway”). Over-representations of pathways related to cancer (chronic myeloid leukemia; renal cell carcinoma) [31] and brain injuries (neurotrophin signaling pathway) [32] were also found in the subnetwork. In all, these results suggest that genetic variants associated with mortality perturb molecular networks involving the immune cells, which may lead to severe disease.

### 2.5. Sepsis-Associated SNPs in Super-Enhancers

**Figure 3:**
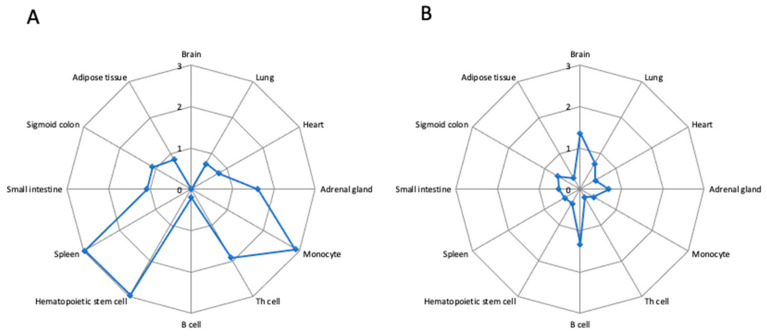
Sepsis-associated SNPs in super-enhancers and typical enhancers. Radar plots show the density of non-coding SNPs within super-enhancers (**A**) and typical enhancers (**B**) in 12 tissues and cell types. The SNP density (SNP/10 MB sequence) was calculated by first counting the number of SNPs within super-enhancers and typical enhancers, which was further divided by the numbers of base pairs of the super-enhancer or typical enhancer region in the same tissue or cell type, and then multiplied by 10 million. The center of the plot is 0, the SNP density (SNP/10 MB sequence) is shown on the respective axis for each tissue or cell type.

Super-enhancers are of particular interest as they modulate the gene expression and are active in tissue or cell type specific manner [33]. We crossed the genomic coordinates of the 139 SNPs with those of enhancer and super-enhancers in 86 tissue or cell types [33]. Figure 3 shows the density of non-coding SNPs associated with mortality in septic shock patients in the super-enhancers and typical enhancers in 12 out of the 86 tissue and cell samples. Moreover, 12.2% of these SNPs (17/139) occurred in the super-enhancers of CD14+ monocytes (Table S5). This led to a density of 2.91 SNPs per 10 MB, whereas the density of the sepsis-associated SNPs was 0.37 SNPs per 10 MB in typical enhancer of monocytes. We further found a significant overlap between SNPs and monocyte super-enhancers using a method based on Monte Carlo simulation (*p* = 0.003). We found 3.44 SNPs within the monocyte super-enhancers after permutating the genomic elements, whereas we identified 17 SNPs within the monocyte super-enhancers. Similarly, an enrichment of the sepsis-associated SNPs was found for Th memory lymphocytes (*p* = 0.015; random overlap = 0.58; observed overlap = 4), CD34+ hematopoietic stem cells (*p* = 0.01; random overlap = 1.68; observed overlap = 5), and spleen (*p* = 0.012; random overlap = 2.98; observed overlap = 12) (Figure 3). As shown in Table S5, we also detected an enrichment for other hematopoietic stem cell samples (*p* = 0.024; random overlap = 1.69; observed overlap = 7) and for naïve Th lymphocytes (*p* = 0.024; random overlap = 0.73; observed overlap = 4). In conclusion, our results show that genetic variants associated with mortality are enriched in monocyte super-enhancers. This suggests that the alteration of gene expression in monocytes may play a central role in the mortality in patients with septic shock.

### 2.6. Prioritization and Annotation of Non-Coding Functional SNPs

**Figure 4:**
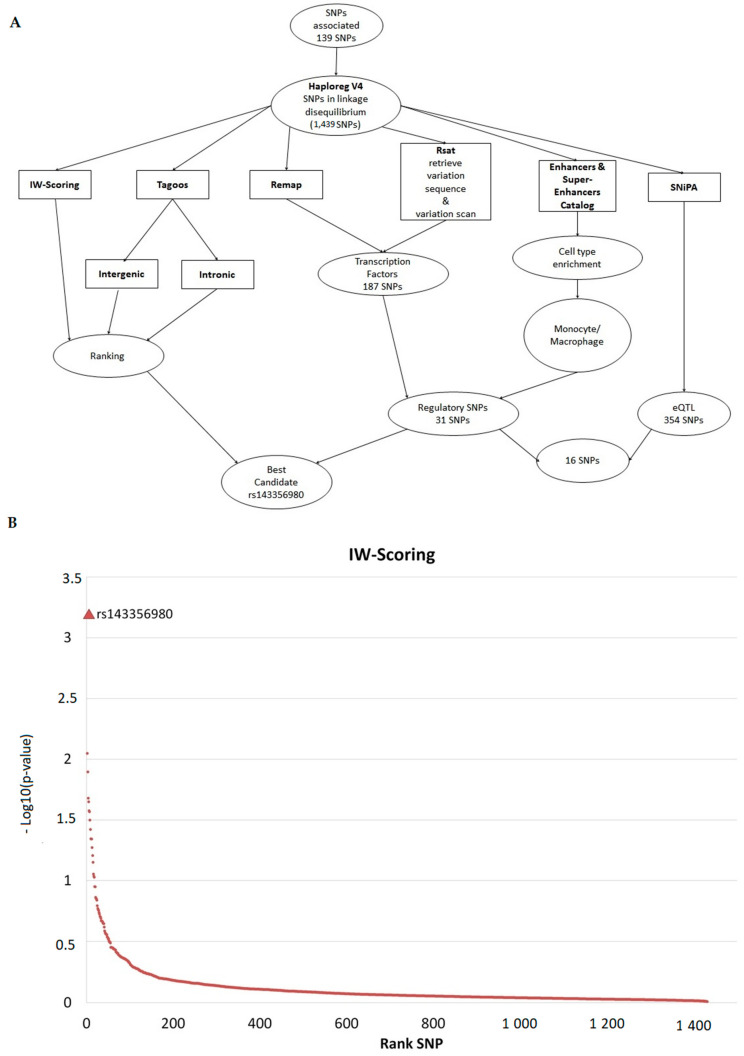
Schematic outline of non-coding SNP annotation and prioritization. (**A**) Schematic outline of non-coding SNP annotation and selection. SNiPA was used for looking for eQTL, whereas RSAT and ReMap were used for identifying transcription factors binding sequences containing the SNPs. Enhancer and super-enhancer annotation was based on the catalog published by Hnisz [33]. IW-scoring and TAGOOS methods were applied to rank the SNPs. (**B**) Prioritization of non-coding SNPs on the basis of IW-scoring method.

We identified 1311 SNPs in strong linkage disequilibrium (r^2^ ≥ 0.8) with the SNPs associated with either early mortality or late mortality (Tables S1 and S2), leading to a list of 1450 SNPs (Table S6). Since 1439 SNPs were in non-coding regions, we further looked for regulatory SNPs (Table S6). Figure 4A shows the outline of SNP annotation and prioritization.

We crossed the genomic coordinates of the 1439 SNPs with those of enhancer and super-enhancers from the catalog published by Hnisz [33]. Among the non-coding SNPs, 505 SNPs were found to be located within enhancers or super-enhancers in at least one of the cell or tissue types. (Table S7). Most of the SNPs were located within super-enhancers of monocytes, spleen, or hematopoietic cells. In particular, 150 SNPs were located within enhancers or super-enhancers in CD14+ monocytes. We further searched for transcription factors that may bind to the sequence containing the SNPs associated with mortality or the SNPs in linkage disequilibrium with them. To this aim, we used the ReMap tool [34], which is a catalog of ChIP-seq results, and regulatory sequence analysis tools (RSAT) [35], which analyzes the sequence containing the SNPs and scans a collection of motifs binding transcription factors. We identified 187 SNPs that may alter the binding of transcription factors (Table S8). Among them, 31 SNPs were located within enhancers or super-enhancers, on the basis of the catalog published by Hnisz et al. [33], whereas 16 SNPs were annotated as expression quantitative trait loci (eQTLs) (Table S9). Among the 16 SNPs, 14 SNPs were located within two super-enhancers located within either the *RNF135* gene locus or the *CISH* and *MAPKAPK3* gene locus. Interestingly, rs2170840 and rs616689 within the *CISH* and *MAPKAPK3* gene locus were associated with late mortality with a *p* value lower than 10^−8^ (Table 1).

In parallel, we ranked the SNPs associated with mortality and the SNPs in linkage disequilibrium with them with two bioinformatic tools, named TAGOOS [36] and integrative weighted (IW) scoring framework [37]. Each tool gives a score based on genomic and epigenomic annotations to predict the regulatory effect of the SNPs. Figure 4B shows the results of the ranking of the SNPs on the basis of the IW-score. The details are in Table S10. Among the SNPs associated with mortality or the SNPs in linkage disequilibrium with them, the SNP with the highest IW-score was rs143356980, which is located close to rs2170840 and rs616689 within the *CISH* and *MAPKAPK3* gene locus on chromosome 3. Noticeably, rs2170840 and rs616689 were ranked in 143th and 23th position by using the IW-scoring method, respectively. rs143356980 was ranked in 13th position on the basis of the intergenic TAGOOS score, whereas it had the best score among the SNPs within chromosome 3. rs2170840 and rs616689 were ranked in 99th and 64th position on the basis of the intergenic TAGOOS score, respectively. Interestingly, rs143356980 was located within a super-enhancer of CD14+, CD4+, CD8+, CD34+, and spleen cells (Table S7), and was in linkage disequilibrium with eQTLs, including rs2170840 and rs616689. Moreover, rs143356980 was in linkage disequilibrium (r^2^ > 0.55) with 13 out of the 14 SNPs associated with late mortality and located within the *CISH* and *MAPKAPK3* gene locus (Table S2). rs143356980 was in strong linkage disequilibrium (r^2^ > 0.80) with 4 SNPs associated with late mortality: rs2239751, rs2239751, rs12492982, and rs17051403 (Table 1).

**Figure 5:**
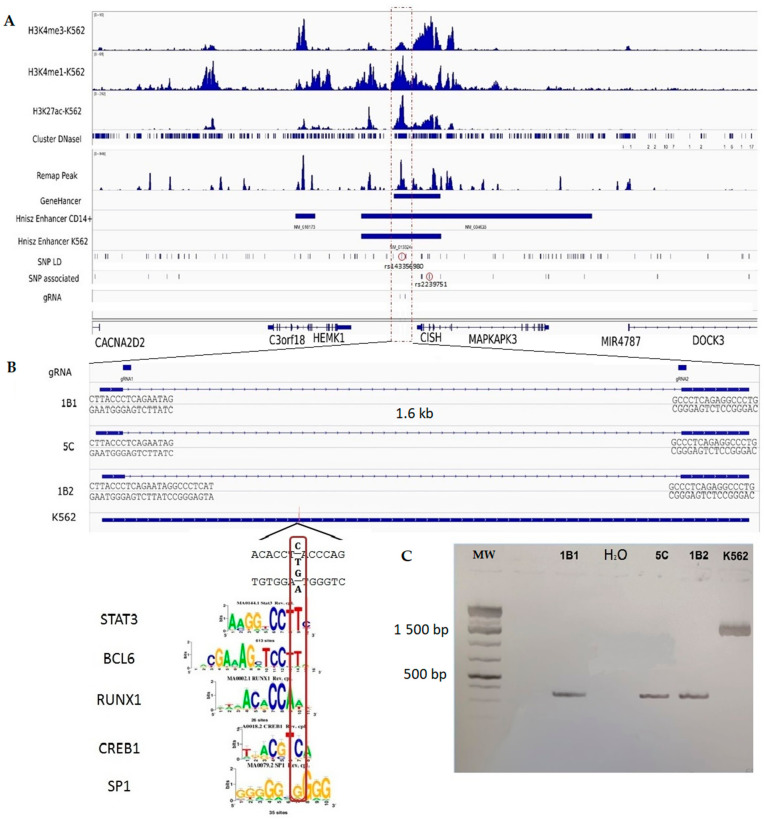
SNPs associated with mortality due to sepsis overlap enhancers in K562 cells and monocytes in the *CISH*-*MAPKAPK3* gene locus. (**A**) Peaks of specific histone marks such as H3K4me3 (active promoters), H3K4me1, H3K27ac (active enhancers), and DNAse I hypersensitivity sites (open chromatin) are shown in K562 cells. Peaks of ChIP-seq for transcription factors from the Remap catalog, and location of enhancer predicted by GeneHancer and super-enhancer in CD14+ monocytes (Hnisz Enhancer CD14+) and in K562 cells (Hnisz Enhancer K562) are also indicated. The genomic position of the SNPs associated with mortality due to sepsis (SNP associated) and that of SNPs in linkage disequilibrium with them (SNP LD) is pointed out. Positions of guide RNA (gRNA) used to generate the deleted K562 cells (1B1, 5C, and 1B2) by CRISPR/Cas9 method are indicated. (**B**) Zoom on the region (framed in (**A**)) containing rs143356980 in K562 WT cells and in 1B1, 5C, and 1B2 K562 deleted cells. Flanking sequences, and the motifs of transcription factors binding sites on the rs143356980 (red box) are specified. (**C**) PCR controls performed on the genomic DNA of WT K562 cells (K562) and in 1B1, 5C, and 1B2 deleted K562 cells using primers located upstream and downstream of the deleted region (MW, molecular weights).

The Figure 5 shows a detailed view of the *CISH* and *MAPKAPK3* gene locus, which contains 14 SNPs associated with late mortality with an FDR of 5% and SNPs in linkage disequilibrium with them. These include rs143356980, which is located within a peak of H3K4me1, a peak of H3K27ac histone mark, a DNAse I hypersensitivity site, and a region binding to several transcription factors. Furthermore, rs143356980 is located within GH03J050580 from the GeneHancer catalog [38], and a super-enhancer of monocytes from the catalog by Hnisz et al. [33]. Moreover, the genomic region containing rs143356980 can be considered as a good enhancer candidate, the activity of which may be altered by rs143356980.

### 2.7. Experimental Validation of the Regulatory Effect of rs143356980

**Figure 6:**
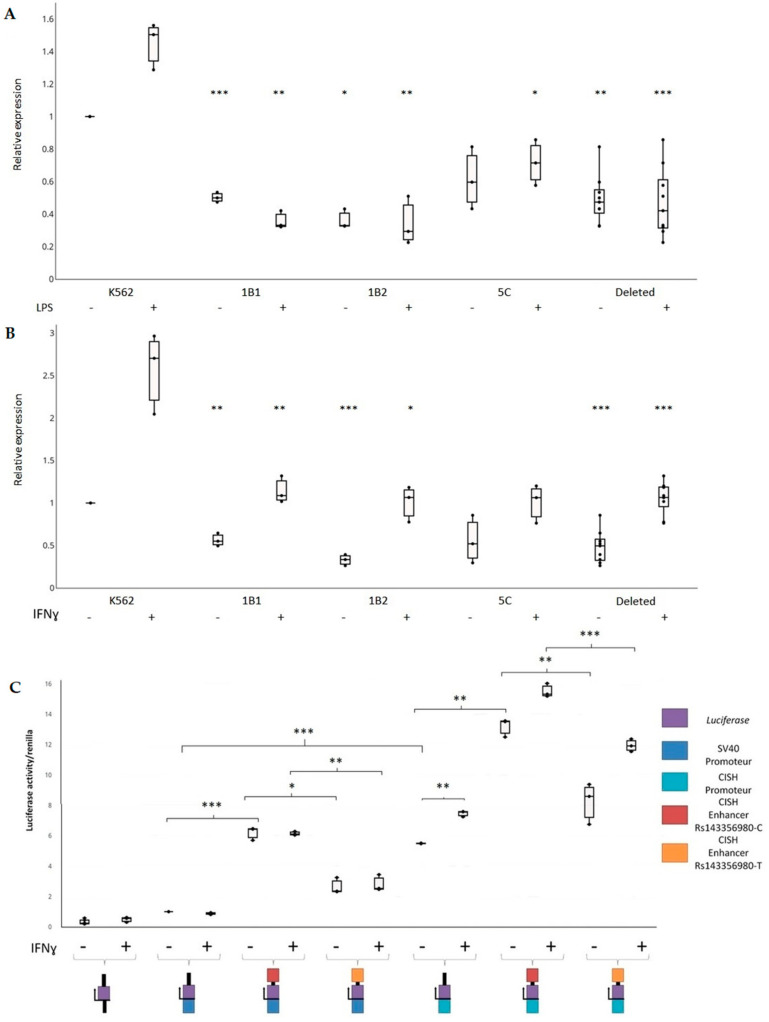
Enhancer effect of the genomic region containing rs143356980 on *CISH* expression. (**A**) *CISH* expression measurements in K562 cells and K562^−/−^ cells either unstimulated (-) or stimulated (+) with LPS. (**B**) *CISH* expression measurements in K562 cells and K562^−/−^ cells either unstimulated (−) or (+) with IFNγγ. For (**A**,**B**), three independent experiments with triplicates were performed, leading to nine measurements for each condition. The box plot of the mean of triplicates is shown for the three experiments. Mixed models that took into account the clustering of triplicates into the experiments were used to compare K562 cells to each K562^−/−^ clone. *p* values are shown for the comparison of the unstimulated K562^−/−^ clones with unstimulated K562 cells and for the comparison of the stimulated K562^−/−^ clones with stimulated K562 cells: *** for *p* < 0.001; ** for *p* < 0.01; * for *p* < 0.05. (**C**) Effect of rs143356980 on the enhancer activity. DNA sequences containing either rs143356980-C allele or rs143356980-T allele were cloned into a luciferase reporter that contained no promoter, a minimal promoter or *CISH* promoter. Three independent experiments with triplicates were performed in K562 cells unstimulated (−) or stimulated (+) with IFNγ, leading to nine measurements for each condition. The box plots show the mean of triplicates for three experiment. Mixed models that took into account the clustering of triplicates into the experiments were used to compare the luciferase activity of the constructs, with a special emphasis of the comparison of constructs containing rs143356980-C allele with those containing rs143356980-T allele. *p* values are shown for unstimulated cells and stimulated cells.

The human erythroleukemia cell line K562 is multipotential, myeloid malignant cells that spontaneously differentiate into progenitors such as erythrocytes granulocyte and monocytic series [39,40]. This cell line is a predilection model in immunology due to their intrinsic properties and their ability to be easily transfected. Interestingly, SNPs associated with late mortality due to sepsis overlap the enhancer marks H3K4me1 and H3K27Ac in K562 cells and monocytes in the *CISH*-*MAPKAPK3* locus (Figure 5A). We then generated K562 homozygous mutated cells, in which a 1636 bp genomic region around rs143356980 was deleted (K562^−/−^) using CRISPR/Cas9 system in order to study the impact of this deletion on gene expression (Figure 5B). Three independent clones were used to measure the expression of *CISH* and to compare it with that of non-deleted K562 cells. The genomic region of interest has been sequenced for wild type cells and mutated cells. The results confirmed the existence of the deleted region of 1636 bp for the 1B1 and 5C clones and that of 1628 bp for the 1B2 clone (Figure 5B,C); these included sequencing results (data not shown). Quantitative real-time PCR assays showed a significant downregulation of *CISH* transcripts in K562^−/−^ cells (*n* = 27) versus non-deleted K562 cells (*n* = 9) with (*t* = 7.90, *p* < 0.001) and without stimulation by IFNγ (*t* = 7.74, *p* < 0.001). These differences remained significant when taking into account the clustering of triplicates into three experiments for K562 and each K562^−/−^ clone (*p* = 0.004 for unstimulated cells, *p* = 0.007 for cells stimulated by IFNγ), as shown in Figure 6B. Similarly, another series of experiments showed a downregulation of *CISH* in K562^−/−^ cells (*n* = 27) versus non-deleted K562 cells (*n* = 9) with (*p* = 0.008) and without stimulation by lipopolysaccharide (LPS) (*p* = 0.011), when taking into account the experiment factor (Figure 6A. In addition, after grouping the series of experiments performed with unstimulated cells, the analysis confirmed the effect of the deletion on *CISH* expression (*p* < 0.001).

When analyzing each clone, *CISH* expression was lower in 1B1 (*n* = 9), 1B2 (*n* = 9), and 5C (*n* = 9) clones than that of non-deleted K562 cells (*n* = 9) on the basis of a Student’s *t* test (t > 4.37, *p* < 0.001). When taking into account the clustering of triplicates into three experiments, the downregulation of *CISH* remained significant in 1B1 (*p* = 0.01 for unstimulated cells, *p* = 0.001 for cells stimulated by IFNγ) and 1B2 (*p* = 0.001 for unstimulated cells, *p* = 0.037 for cells stimulated by IFNγ) clones versus non deleted K562 cells (Figure 6B), whereas it was not significant for 5C clone (*p* = 0.054 for unstimulated cells, *p* = 0.114 for cells stimulated by IFNγ). Another series of experiments confirmed this result in unstimulated 1B1 (*p* < 0.001), 1B2 (*p* = 0.003), and 5C clones (*p* = 0.075) (Figure 6A), and provided evidence of a downregulation of *CISH* in cells stimulated by LPS (*p* = 0.006 for 1B1, *p* = 0.010 for 1B2, *p* = 0.028 for 5C). The downregulation of *CISH* in the unstimulated 5C clone was significant when grouping all the experiments (*p* = 0.006), whereas it was highly significant for 1B1 (*p* < 0.001) and 1B2 (*p* < 0.001).

We further cloned upstream a 607 bp region surrounding rs143356980 into a luciferase reporter vector to test its regulatory effect (Figure 6C). The enhancer activity of the region surrounding rs143356980 was detected in cells with the minimal promoter (*t* > 8.9, *p* < 0.001) and cells with the *CISH* promoter (*t* > 2.7, *p* < 0.016), as shown in Figure 6C. This was further confirmed, when taking into account both the experiment factor and rs143356980 allele for the minimal promoter and the *CISH* promoter (*p* < 0.001). Nevertheless, the results showed a clear effect of rs143356980 on the enhancer activity. Moreover, the luciferase activity in K562 cells with both the *CISH* promoter and the enhancer with rs143356980-C allele was significantly higher than that in K562 cells with both the *CISH* promoter and the enhancer with rs143356980-T allele (*p* = 0.004 for unstimulated cells and *p* < 0.001 for cells stimulated with IFNγ), after taking into account the clustering of triplicates into three experiments. Similar results were obtained in K562 cells with the minimal promoter (*p* = 0.023 for unstimulated cells and *p* = 0.010 for cells stimulated with IFNγ). Interestingly, there was no effect of IFNγ on the luciferase activity in K562 cells with the minimal promoter (Figure 6C). In contrast, the luciferase activity in K562 cells with the *CISH* promoter was higher in cells stimulated with IFNγ than that in unstimulated cells (*p* < 0.003), indicating that IFNγ acts on the *CISH* promoter.

Overall, our results show that the genomic region of interest has an enhancer activity that is perturbed by rs143356980. The effect of the variants on the activator activity and further on the regulation of cytokines could partly explain the transition from mild to severe sepsis in some patients.

## 3. Discussion

In this study, we assessed the association of SNPs with early and late mortality in septic shock patients at the genome level, and we looked for biological pathways that could be disrupted by genetic variation. We then annotated and prioritized the SNPs associated with mortality and the SNPs in linkage disequilibrium with them and characterized the functional significance of the best candidates.

This present GWAS follows a previous study using the same PROWESS cohort [18], but designed after removal of the patients in the aPC arm. The next randomized clinical trial PROWESS SHOCK failed to show a benefit of aPC on mortality, motivating the immediate removal of aPC from the market. As a consequence, this aPC failure to reduce mortality in septic shock allowed to use the shocked patients from both placebo and aPC arms of the PROWESS cohort to perform the GWAS, a selection that differed from the previous GWAS [18]. The treatment with aPC was then considered only as a covariate. The growing evidence for potential differing mechanisms for early versus late death [19,20] was then considered to test SNP associations. The early stimulation of inflammation processes appears to be rapidly followed by a downregulation of these processes through dominant anti-inflammatory patterns, which can be considered suitable for maintaining the tissue fitness and reducing the risk of death [41]. If it persists over time, such acquired immunosuppression may be associated with higher risk of secondary infection [42]. The present GWAS allowed us to identify SNPs associated with mortality in septic shock patients.

Based on an FDR of 5%, 32 and 107 SNPs were associated with early and late mortality, respectively. These associations reduced to 12 and 16 SNPs after the Bonferroni correction for early and late mortality, respectively. Individuals having four or more risk alleles had a 12-fold higher or a 123-fold higher risk of death than those without risk alleles for early and late death, respectively. For early death, the strongest associations between intra-genic SNPs were located within *CYP11B2*, a gene encoding aldosterone synthase, a key enzyme of the aldosterone biosynthesis. Variants of such a gene have never been reported to be associated with human shock status and/or severe infection, but have been shown to be largely associated with hypertension and atrial fibrillation [43]. The other important SNPs associated with early mortality were located within *PTPN11*, which is also known as *SHP2*. The proteins encoded by this gene are members of the protein tyrosine phosphatase (PTP) family that are known to be signaling molecules regulating a variety of cellular processes. This PTP family contains two *tandem Src homology-2 domains*, which function as phospho-tyrosine binding domains and mediate the interaction of the PTP with their substrates. The protein encoded by *PTPN11* is implicated in reduced JAK/STAT signaling when it is elevated, which may reduce MHC expression induced by INFγ [31]. SHP2 activation induced by human CMV infection is responsible for the downregulation of INFγ-induced STAT1 tyrosine phosphorylation [44]. In addition, the PD1/PDL1 interaction has been shown to inhibit T cell receptor signaling by recruiting SHP1/2 phosphatases [45]. For late mortality, it should be noticed that *FER* reported to be associated with mortality in sepsis caused by pneumonia [15] was associated with mortality in septic patients in our study on the basis of an FDR of 5%. FER that is a protein tyrosine kinase acting downstream of cell-surface receptors, has been shown to influence leucocyte recruitment in response to LPS [46] to inhibit neutrophil chemotaxis [47], and to alter the endothelial response to LPS stimulation [48]. Furthermore, the strongest associations were found within cytokine-inducible SRC homology 2 (SH2) domain protein (*CISH*) and *MAPKAPK3*. *MAPKAPK3* is involved in the MAP Kinase pathway, which is known to regulate the activation of immune cells. *CISH* is the first member of the suppressor of cytokine signaling (SOCS) family. An association has been shown between *CISH* polymorphisms and susceptibility to infectious diseases including malaria, bacteremia or tuberculosis [25]. In addition, rs414171-T allele that was associated with susceptibility to bacteremia, tuberculosis and malaria has been shown to reduce the promoter activity of *CISH* and its expression in human PBMCs after stimulation by IL-2 [25,49]. Since CISH is known to suppress STAT5 in T cells, it has been proposed that decreased levels of *CISH* lead to enhanced activation of STAT5 and enhanced activation of Treg lymphocytes, and as a consequence, a suppressed immune response against bacteria and other pathogens [25].

Noticeably, the *CISH* locus was highlighted by our bioinformatic analyses, which aimed to annotate and prioritize SNPs associated with mortality and the SNP in linkage disequilibrium with them. Since more than 95% of the SNPs associated with mortality or the SNPs in linkage disequilibrium with them were located in non-coding regions, we hypothesized that the vast majority of the causal genetic variants are regulatory variants. More generally, most of the GWAS variants are non-coding, emphasizing the potential role of regulatory variants in complex diseases [50,51]. Moreover, we investigated 1439 non-coding SNPs including SNPs associated with mortality and SNPs in linkage disequilibrium with them. Among those SNPs, rs143356980 was the best candidate using the IW-scoring method and was ranked in 13th position on the basis of the intergenic TAGOOS score. Interestingly, it is located near the *CISH* gene, and is in strong linkage disequilibrium with four SNPs highly associated with late mortality in patients with septic shock; these includes rs2239751, which has been also associated with tuberculosis [52,53], and persistent hepatitis B virus infection [54]. Furthermore, rs143356980 is located within a super-enhancer for monocytes and T lymphocytes, according to the database by Hnisz et al. [33]. Using the CRISPR-Cas9 genome editing method, we showed that the sequence containing rs143356980 has an enhancer activity on the *CISH* gene in unstimulated K562 cells and K562 cells stimulated with either LPS or IFNγ. Using the luciferase reporter assay, we showed the effect of rs143356980 on the enhancer activity in unstimulated K562 cells and K562 cells stimulated with IFNγ. More specifically, rs143356980-T decreased the enhancer activity compared to rs143356980-C allele. In all, these results suggest that genetic variation within the enhancer containing rs143356980 influences *CISH* gene expression, Jak/Stat signal transduction, and the risk of death in septic shock patients. It is not excluded, nevertheless, that other SNPs within the same enhancer or other regulatory regions alter *CISH* gene expression and susceptibility to sepsis. These include rs414171, which has been shown to reduce *CISH* expression in human PBMCs after stimulation by IL-2 [25,49]. rs143356980 and other genetic variants in an enhancer may act through the same mechanisms, leading to susceptibility to sepsis.

Since the expression of *CISH* is induced through the stimulation of other receptors, genetic variation altering *CISH* expression may have functional consequences in other cells. *CISH* expression is induced in response to EPO, IL-2, IL-3, IL-5, GM-CSF in hematopoietic cells, leading mostly to the activation of STAT5 [55,56]. In addition, *CISH* is an inducible gene in NK cells stimulated by IL-15, and deletion of *CISH* increased proliferation, IFNγ production and cytotoxicity against tumors [57]. Since NK cells in septic patients have been shown to produce low levels of IFNγ and to have a decreased cytotoxicity activity [58], low levels of *CISH* may influence susceptibility to sepsis through an NK cell dependent mechanism. *GM-CSF* expression is induced in macrophages infected by *M. tuberculosis*, leading to *CISH* expression and an increased replication of *M. tuberculosis* [59]. Moreover, LPS and IFNγ induce the expression of *CISH* in human monocytes, as shown in a transcriptomic study [60]. Similarly, we report in the present study an increase of the *CISH* expression in K562 cells stimulated either by LPS or IFNγ. The functional effect of *CISH* expression levels remains, however, to be investigated in monocytes or macrophages in septic patients.

Forty-five genes that encode proteins contained the SNPs associated with early or late mortality or the SNPs in linkage disequilibrium with the SNPs. Enrichment in biological pathways (Kyoto encyclopedia of genes and genomes-KEGG and BIOCARTA) was used to investigate the involved underlying biological functions. Since no significant enrichment based on the 45 genes was found, we mapped the proteins encoded by these 45 genes on a high-quality protein–protein interaction network [30]. Thirty proteins out of the 45 proteins were included in the whole protein–protein network, leading to identify a sub-network that contains 27 proteins associated with mortality and their 298 direct interactors. For example, CISH and PTPN11 shared five direct interactors, whereas CISH and FER shared one interactor. This suggests that genetic variants altering the function or the expression of proteins belonging to the sub-network may act in combination to influence mortality in septic shock patients. Furthermore, this subnetwork was enriched for 79 significant pathways, including Toll-like receptor, IL-6, Jak-STAT, and T cell receptor signaling pathways as well as aldosterone-regulated sodium reabsorption. Thus, the dysregulation of the renin-angiotensin-aldosterone system and the dysregulation of the monocyte/macrophage activation or the T-cell activation may be involved in sepsis-induced associated organ failure. In the same way, sepsis-associated SNPs were enriched in the super-enhancers of adrenal gland that produces aldosterone; furthermore, sepsis-associated SNPs were highly enriched in the super-enhancers of monocytes and Th lymphocytes. Moreover, Davenport et al. recently reported that patients with higher early mortality had an increased expression of negative regulators of TLR signaling, and a downregulation of human leucocyte antigen class II genes and most genes implicated in T cell activation [61]. The clinical relevance of these findings is strongly supported by the significant benefits of associating clinical traits with SNPs to predict early and late death [62] (Figure 1D,E).

Our results suggest that genetic variations in different genes including *CISH* alter the activation of immune cells and, in turn, increase the risk of both early and late mortality in patients with septic shock. To provide greater homogeneity to our GWAS study, only European patients with septic shock were selected. This allowed us to look for SNP in strong linkage disequilibrium with SNPs associated with mortality, and to annotate them for their molecular function. Furthermore, we looked for the potential functional significance of the identified SNPs using protein–protein interactions and bioinformatics tools predicting regulatory SNPs. Finally, we performed experimental studies confirming the regulatory effect of a bona fide candidate SNP.

In conclusion, this GWAS analysis identified new loci relevant for mortality in European patients with septic shock. Here, we provide evidence for (i) different covariates and SNPs that influence early or late mortality, supporting the concept to separate early and late mortality; (ii) different SNPs strongly associated either with early or with late mortality; (iii) a protein–protein sub-network highlighting biological pathways, such as the Jak/stat pathway; (iv) the combination of clinical traits and SNPs may better predict early and late mortality; (v) a regulatory effect of a sequence containing candidate SNPs on *CISH* expression, and a high effect of rs143356980 on the enhancer activity suggests an important role of this region on the immune response modulation in patients. However, independent GWAS testing the same SNPs or replication studies focused on the same phenotypes in septic shock patients are required to confirm our association results. Further studies depicting the effect of the transcription level of *CISH* on the intensity of the immune response in monocytes/macrophages, crucial during sepsis development are needed.

## 4. Materials and Methods

### 4.1. Patients, Database, and Study Design

The flow chart of the study shown in Figure 1A is also shown in Figure S1, providing all steps and reasons for the final size of the cohort used for GWAS. The studied cohort was kindly provided through a formal contract between Eli Lilly and Company (Eli Lilly and Company; Indianapolis, IN, USA), the owner of the PROWESS database, and the senior author of the present study (DP). The RCT PROWESS was a multi-center, randomized, double-blind, placebo-controlled study evaluating the efficacy of activated protein C (aPC) in severe sepsis. The bioethics committees for each study center approved the trial protocol and written consent was obtained from all participants or their next of keen. The DNA collection was included in the trial with the intent of testing for factor V polymorphisms, and consultation with bioethics committees confirmed that no additional consent was necessary for further genetic investigations on anonymized samples. The entry criteria and clinical phenotyping for the PROWESS study have previously been reported [63].

The recent report of the RCT PROWESS SHOCK [3] showed that aPC treatment did not improve outcome in septic shock patients in comparison with the placebo group. This allowed us to further investigate the PROWESS database, selecting exclusively septic shock patients, and pooling placebo and treated individuals. aPC treatment was, nevertheless, included as a covariate and was tested for the studied phenotypes. The patients who were selected in the present study do meet the sepsis-3 definition [64], having at least two or more organ failures.

The clinical characteristics, co-morbidity presence, and day 1 plasma IL-6 levels as a marker of systemic inflammation [65] were collected (Table 4). Because different mechanisms may drive early mortality compared to late mortality (after 7 days) [20,66], the association with outcome was separated into early (before day 7) and late (between day 7–day 28) death.

### 4.2. Cell Line and Culture Conditions

The chronic myelogenous leukemia cell line K562 (CCL-243) was obtained from the American Type Culture Collection (ATCC, Manassas, VA, USA) and maintained in RPMI (Sigma, St. Louis, MO, USA, L9143) supplemented with 20% FBS (Gold, PAA) at 37 °C and 5% CO_2_. For cell stimulation, 10^6^ K562 cells were incubated with IFN-γ (Miltenyi, Bergisch Gladbach, Germany, 130-096-484) at 100 ng/mL for 6 h or LPS (Sigma, St. Louis, MO, USA, L9143) at 100 ng/mL for 24 h.

### 4.3. Single Nucleotide Polymorphism (SNP) Selection

Briefly, genomic DNAs were pre-amplified using a GenomePlex whole genome amplification kit from Sigma-Aldrich (St. Louis, MO, USA). An Illumina Human 1M-Duo BeadChip (Illumina, San Diego, CA, USA) was used for genotyping as previously reported [18]. Thus, 1,199,187 SNPs were genotyped for each individual. The SNPs were selected according to minor allelic frequencies, call rates and the Hardy–Weinberg equilibrium. As a first step for quality control, we applied “check.marker” based method implemented in the R package GenABEL [67] to assess the call rate for the SNPs. The SNPs with minor allele frequencies below 1% and genotyping rates below 95% were removed from the dataset, resulting in 948,573 SNPs. The individuals with a call rate below 95% were excluded from the analyses, resulting in 1411 individuals (Figure 1A and Figure S1).

The individuals were further selected on the basis of a population stratification analysis. First, a multidimensional scaling using Euclidean distances (principal component analysis) was applied using the cmdscale method implemented in the “stats” package [68]. Second, the “kmean” partitioning algorithm (R stats package) was used [68]. This resulted in three clusters, the largest one bringing together essentially CEU individuals. The two other clusters were composed of 122 and 113 individuals, respectively. Thus, 1176 individuals comprising the largest cluster were kept for further analysis (Figure S1). A departure from the Hardy–Weinberg expectation was assessed and deviating SNPs (*p* < 0.05) were excluded, resulting in 896,358 SNPs. The genotype rate was then re-evaluated, and individuals displaying more than 5% of the missing genotypes were excluded, resulting in 1173 individuals. Within this cohort, only patients who underwent septic shock were selected for the association analyses (*n* = 832) (Figure 1A and Figure S1).

### 4.4. Association Analyses and Statistical Methods

The clinical characteristics, presence of comorbidities and levels of IL-6 in the plasma (taken as a marker of systemic inflammation) [65] were recorded (Table 4). The plasma levels of IL-6 measured at day 1 were expressed in logarithm base 10 due to scattered values. The organ components of the sequential organ failure assessment (SOFA) score were provided by the PROWESS dataset. The SOFA score that is an evaluation of the severity is based on scores reflecting the function of the respiratory, cardiovascular, hepatic, coagulation, renal, and neurological systems. Table 4 shows statistics for all the European patients with septic shock, and for the deceased and surviving patients before and after 7 days. A logistic regression analysis was performed to assess the association of (i) early or (ii) late mortality with covariates (Table 5), using the glm function from the R software. Covariates with *p*-values below (or equal) to 0.2 were selected by univariate analysis and further included in the multivariate logistic regression model. The best model was chosen on the basis of the Akaike Information Criterion in a backward and forward stepwise procedure [69]. Only covariates having a *p*-value below or equal to 0.05 in the model were used for the genetic association analyses (Table 5). The significant covariates were taken into account in further analyses.

The GenABEL package [67] was used for the GWAS, assuming an additive mode of inheritance. It allowed us to perform analyses with adjustment for covariates. The score test in the “qtscore” function of GenABEL was applied, and yielded a *p*-value for each SNP, after correcting for the inflation factor lambda. The Bonferroni correction and false discovery rate (FDR) procedure were used to correct for multiple tests in each association study. The nominal *p*-value corresponding to a genome-wide risk of 5% was 5.58 × 10^−8^ on the basis of the Bonferroni correction. In addition, we used the FDR method that controls the expected proportion of false positives among associations considered significant. To this aim, we used the qvalue R library with a threshold set to 5% [67]. The Manhattan plots for GWAS results were obtained using the GenABEL R library; Manhattan plots show −log10 of the *p*-values along all autosomes (Figure 1B,C). Finally, odds ratios and 95% confidence intervals were calculated using the “glm” function in R for each significant SNP and for the SNP groups. The adjusted odds ratios that take into account the effect of the covariates were calculated on this basis. The unadjusted odds ratios were also calculated without including the covariates in the logistic regression model.

We used the Haploreg database to identify the SNPs in linkage disequilibrium with the SNPs associated with mortality [70]. The linkage calculation was based on the 1000 genome project data restricted to European individuals, and the SNPs with r^2^ > 0.8 were identified. This led to a determination of the chromosomal region that likely contains the causal SNP.

The receiver operating characteristic (ROC) curves of a logistic regression model were plotted using the epicalc R package. For the genetic part, the leader SNP of each associated locus (the SNP with the lowest q-value for each locus) was included in the logistic regression model. The significance of the difference between the ROC curves was assessed by the likelihood ratio test.

Student’s *t* test was used to assess on the one hand the effect of CRISPR-Cas9-mediated deletion of enhancer on *CISH* expression in K562 cells, and on the other hand that of rs143356980 on reporter gene expression in K562 cells. For each type of cells (non-deleted or deleted K562 cells) and each condition, three experiments were performed with triplicates. Moreover, mixed linear models were further used to take into account the triplicates within each of the three experiments.

### 4.5. Protein–Protein Network and Functional Enrichment

The products of the genes associated with the two phenotypes were mapped on a high quality human interactome network containing 74,388 binary protein–protein interactions between 12,865 proteins [30]. Their first neighbors in the network and their interactions were subsequently retrieved. Then, we searched for a subnetwork based on the interaction between proteins associated with mortality and their direct interactors. The associated proteins separated by more than one interactor were discarded. The functional annotation was performed using the DAVID web tool [71], and the whole interactome was used as background. The significance of enrichments was computed for each term of the KEGG [72] and BIOCARTA pathways [73], on the basis of a FDR of 5%.

### 4.6. SNP Annotation and Prioritization

SNPs in linkage disequilibrium with SNPs associated with early or late death were identified with haploregV4 [70]; a threshold of r^2^ ≥ 0.8 was used in the CEU population. ReMap and RSAT were employed to evaluate the effective transcription factor binding to the sequence containing the SNP. ReMap integrates the results of transcriptional regulators ChIP-seq experiments from both Public and Encode datasets [34]. ReMap allowed us to cross our genomic regions against the ReMap catalog of transcription factor binding peaks. Regulatory Sequence Analysis Tool (RSAT) was used to explore the sequences containing the SNPs of interest [35]. Variation-scan that is a RSAT tool was used to assess the potential effect of the SNP on transcription factor binding and to identify motifs that may be affected by the SNP. A catalog of super-enhancers in a broad range of human cell types was used to identify cells or tissues, for which super-enhancers contained SNPs of interest [33]. The significance of the overlap between SNPs and super-enhancers was assessed using OLOGRAM, which considers the number of overlapping base pairs with a shuffling method conserving inter-region length [74]. The SNipa database was used to look for eQTLs [75].

To prioritize all the candidate non-coding SNPs, integrative bioinformatics approaches was used. TAGOOS that uses a supervised machine-learning algorithm was employed to classify the potential regulatory SNPs on the basis of a broad range of annotations such as epigenomic marks or eQTLs [36]. IW-scoring that integrates scores from 11 tools was also used to prioritize the candidate SNPs [37].

### 4.7. Genome Editing Using the CRISPR-Cas9 Method

Two gRNAs were designed for each end of the targeted region using the CRISPRdirect tool [76], as shown in Figure 5B. The gRNAs were cloned into a gRNA cloning vector (Addgene, Watertown, MA, USA, 41824) as previously described [77]. The sequences of the forward gRNA and reverse gRNA were CCTCATCAGATAACCTCCAG and ATAGCCCTCAGAGGCCCTGC, respectively. One million cells were transfected with 1 μg of the hCas9 vector (Addgene, 41815) and 1 μg of each gRNA using the Neon Transfection System (Thermo Fisher Scientific, Waltham, MA, USA). Three days after transfection, transfected cells were plated in 96-well plates at limiting dilution (0.5 cells per 100 μL per well) for clonal expansion. Individual cell clones were screened for homologous allele deletion by direct PCR using Phire Tissue Direct PCR Master Mix (Thermo Fisher Scientific), according to the manufacture’s protocol. Forward and reverse primers were designed bracketing the targeted regions to detect deleted and non-deleted clones: GGCTCATTCCCTTGGTCCAG for the forward primer and GCCACTCTCCAACCACTCTG for the reverse primer. Sequencing based on the Sanger method was used to check successful deletion of the sequence of interest.

### 4.8. cDNA Synthesis and qRT-PCR

Total RNA was extracted using RNeasy Plus Mini Kit (Qiagen, Hilden, Germany). 500 ng of RNA was reverse transcribed into cDNA using Superscript VILO Master Mix (Thermo Fisher Scientific). Real-time PCR was performed using Power SYBR Master Mix (Thermo Fisher Scientific) on a QuantStudio 6 Flex Real-Time PCR System apparatus. Forward and reverse primer sequences were AGAGAGTGAGCCAAAGGTGC and TCTTCTGCAGGTGTTGTCGG, respectively. Gene expression was normalized to that of *GAPDH*, as an endogenous control. Relative expression was calculated by the ΔΔ*C*_T_ method, and all data shown were reported as a fold change over the control.

### 4.9. Gene Reporter Assays

*CISH* promoter sequence and the genomic region flanking rs143356980 was amplified from human genomic DNA. Three constructions were cloned into PGL3 vector. First, *CISH* promoter sequence (1573 pb) was cloned upstream of the luciferase gene at the MluI–XhoI sites in pGL3-basic vector. Second, *CISH* enhancer sequence (619 pb) containing rs143356980 was cloned in pGL3-promoter at the BamHI-SalI site. Third, *CISH* promoter and *CISH* enhancer sequence (619 pb) containing rs143356980 were cloned in the previous places in the pGL3-basic vector. Site directed mutagenesis was used to generate the rs143356980 mutation C -> T with the Q5^®®^ Site-Directed Mutagenesis Kit (NEB, Ipswich, MA, USA). A total of 1 × 10^6^ K562 cells were cotransfected with 1 μg of each tested construct and 200 ng of *Renilla* vector using the Neon Transfection System (Thermo Fisher Scientific). Electroporation conditions for K562 cells are described in the CRISPR–Cas9 genome editing section. Six hours after transfection, luciferase activity was measured using the Dual-Luciferase Reporter Assay kit (Promega, Madison, WI, USA) on a Victor Nivp (PerkinElmer). For all measurements, firefly luciferase values were first normalized to *Renilla* luciferase values (controlling for transfection efficiency and cell number). Data are represented as the fold increase in relative luciferase signal over the pGL3-Promoter vector.

## Figures and Tables

**Table 1:** Loci associated with the mortality due to sepsis at day 7 or at day 28.

SNP	CHR: Position	Alleles (MAF)	Risk Allele	*p* Value (q Value)	Unadjusted OR (Adjusted OR)	LD Region r2 > 0.8	Genes Containing SNP	Genes in LD	Phenotype Associated
rs16857698	3: 145685067	A > G (0.014)	G	1.75 × 10^−9^ (7.53 × 10^−4^)	3.52 (4.51)	3:145665563-145685067			D7
rs5029231	3: 145701146	C > T (0.019)	T	1.37 × 10^−8^ (1.73 × 10^−3^)	3.54 (3.99)	3:145686379-145759412			D7
rs6763296	3: 145709314	T > C (0.018)	C	2.55 × 10^−9^ (7.53 × 10^−4^)	3.93 (4.67)	3:145686379-145759412			D7
rs16857836	3: 145752473	G > T (0.014)	T	5.51 × 10^−10^ (4.89 × 10^−4^)	3.79 (5.20)	3:145686379-145759412			D7
rs4544	8: 143994806	T > C (0.010)	C	8.86 × 10^−9^ (1.31 × 10^−3^)	3.24 (6.87)	8:143983592-144018027	CYP11B2	GML	D7
rs11991278	8: 144001245	C > T (0.010)	T	8.48 × 10^−9^ (1.31 × 10^−3^)	3.23 (6.87)	8:143983592-144018027	CYP11B2	GML	D7
rs6981918	8: 144007939	C > A (0.010)	A	8.74 × 10^−9^ (1.31 × 10^−3^)	3.22 (6.85)	8:143983592-144018027	CYP11B2	GML	D7
rs956727	9: 86846933	A > G (0.009)	G	3.22 × 10^−8^ (2.60 × 10^−3^)	4.85 (17.43)	9:86814655-86862104	SLC28A3		D7
rs7974468	12: 112927208	G > A (0.013)	A	1.60 × 10^−8^ (1.78 × 10^−3^)	3.10 (3.34)	12:112819245-112985734	PTPNN11	RPH3A	D7
rs10849640	12: 119712137	G > A (0.116)	A	3.22 × 10^−8^ (2.60 × 10^−3^)	1.65 (2.25)	12:119712137-119725314			D7
rs10849641	12: 119721354	C > T (0.115)	T	2.65 × 10^−8^ (2.60 × 10^−3^)	1.67 (2.27)	12:119712137-119725314			D7
rs10849642	12: 119725314	C > T (0.117)	T	4.04 × 10^−8^ (2.99 × 10^−3^)	1.62 (2.25)	12:119712137-119725314			D7
rs12491812	3: 50556581	C > T (0.011)	T	4.18 × 10^−11^ (1.25 × 10^−5^)	5.62 (7.32)	3:50534635-50645413	CACNA2D2	C3orf18, HEMK1, CISH	D28
rs2239753	3: 50645158	T > C (0.011)	C	2.80 × 10^−11^ (1.25 × 10^−5^)	4.97 (7.02)	3:50555933-50645413	CISH	C3orf18, HEMK1, CACNA2D2	D28
rs2239752	3: 50645413	C > T (0.011)	T	5.43 × 10^−10^ (4.86 × 10^−5^)	4.32 (5.62)	3:50555933-50645413	CISH	C3orf18, HEMK1, CACNA2D2	D28
rs2239751	3: 50647888	A > C (0.011)	C	5.21 × 10^−10^ (4.86 × 10^−5^)	4.32 (5.62)	3:50531386-50875635	CISH	C3orf18, HEMK1, CACNA2D2, MAPKAPK3, DOCK3	D28
rs743753	3: 50651395	C > T (0.011)	T	5.21 × 10^−10^ (4.86 × 10^−5^)	4.32 (5.62)	3:50531386-50875635	MAPKAPK3	C3orf18, HEMK1, CACNA2D2, CISH, DOCK3	D28
rs616689	3: 50668532	G > A (0.014)	A	1.87 × 10^−10^ (3.35 × 10^−5^)	5.09 (5.79)	3:50647343-50751643	MAPKAPK3	CISH, DOCK3	D28
rs9879397	3: 50685642	G > A (0.012)	A	8.79 × 10^−9^ (6.57 × 10^−4^)	4.00 (4.86)	3:50647343-50751643	MAPKAPK3	CISH, DOCK3	D28
rs2170840	3: 50686517	A > C (0.014)	C	1.87 × 10^−10^ (3.35 × 10^−5^)	5.09 (5.78)	3:50647343-50751643	MAPKAPK3	CISH, DOCK3	D28
rs12492982	3: 50698155	C > T (0.011)	T	4.18 × 10^−11^ (1.25 × 10^−5^)	4.85 (7.32)	3:50531386-50875635	MAPKAPK3	C3orf18, HEMK1, CACNA2D2, CISH, DOCK3	D28
rs2035484	3: 50721892	A > G (0.011)	G	5.21 × 10^−10^ (4.86 × 10^−5^)	4.32 (5.62)	NA	DOCK3		D28
rs17051403	3: 50751643	C > A (0.011)	A	5.21 × 10^−10^ (4.86 × 10^−5^)	4.32 (5.62)	3:50531386-50875635	DOCK3	C3orf18, HEMK1, CACNA2D2, CISH, MAPKAPK3	D28
rs17072628	3: 65229760	G > A (0.012)	A	8.25 × 10^−9^ (6.57 × 10^−4^)	3.88 (4.96)	3:65214495-65241577			D28
rs7840669	8: 89929277	A > G (0.015)	G	2.38 × 10^−8^ (1.53 × 10^−3)^	4.23 (3.77)	8:89901960-90133835			D28
rs7953683	12: 79993704	C > T (0.024)	T	3.07 × 10^−8^ (1.72 × 10^−3^)	2.06 (2.07)	12:79919466-80080618	PAWR		D28
rs1502522	17: 51544776	A > G (0.029)	G	2.57 × 10^−8^ (1.53 × 10^−3^)	3.24 (2.64)	17:51519876-51590268			D28
rs1393467	17: 51560869	T > C (0.029)	C	2.57 × 10^−8^ (1.53 × 10^−3)^	3.24 (2.64)	17:51519876-51590268			D28

**Table 2:** Adjusted and unadjusted OR for cumulative effect of allele risk at leader SNPs.

	Early Death		Late Death	
Nb of Risk Alleles	Non-Adjusted OR	Adjusted OR	Non Adjusted OR	Adjusted OR
0	1	1	1	1
1	1.56	1.44	4.75	3.53
2	1.71	1.58	7.95	7.82
3	3.38	3.48	17.96	17.86
≥4	9.40	12.33	70.75	123.35

**Table 3:** List of the proteins associated with mortality.

HGNC Symbol	UniProt Symbol	Presence in the Interactome	Presence in the Sub-Network
ADAP2	ADAP2_HUMAN	yes	yes
ANKFN1	ANKF1_HUMAN	no	no
ANKH	ANKH_HUMAN	yes	yes
ARIH1	ARI1_HUMAN	yes	yes
ASIC2	ASIC2_HUMAN	yes	yes
ATAD5	ATAD5_HUMAN	yes	yes
C3orf18	CC018_HUMAN	no	no
C6orf170	BROMI_HUMAN	no	no
CACNA2D2	CA2D2_HUMAN	no	no
CISH	CISH_HUMAN	yes	yes
CRLF3	CRLF3_HUMAN	yes	yes
CYP11B2	C11B2_HUMAN	no	no
DOCK3	DOCK3_HUMAN	yes	yes
DPYD	DPYD_HUMAN	yes	yes
EHMT1	EHMT1_HUMAN	yes	yes
FER	FER_HUMAN	yes	yes
GML	GML_HUMAN	no	no
GPR158	GP158_HUMAN	yes	yes
GREM2	GREM2_HUMAN	yes	no
HECTD4	HECD4_HUMAN	no	no
HEMK1	HEMK1_HUMAN	yes	yes
IFIT1B	IFT1B_HUMAN	no	no
KPTN	KPTN_HUMAN	yes	yes
LBP	LBP_HUMAN	yes	yes
LIPA	LICH_HUMAN	no	no
MAPKAPK3	MAPK3_HUMAN	yes	yes
NAPA	SNAA_HUMAN	yes	yes
NCKAP5	NCKP5_HUMAN	yes	yes
NLN	NEUL_HUMAN	no	no
OSCP1	OSCP1_HUMAN	no	no
PAWR	PAWR_HUMAN	yes	yes
PPFIA2	LIPA2_HUMAN	yes	yes
PTPN11	PTN11_HUMAN	yes	yes
RBFOX1	RFOX1_HUMAN	yes	yes
RPL6	RL6_HUMAN	yes	yes
RNF135	RN135_HUMAN	yes	yes
SLC15A1	S15A1_HUMAN	yes	no
SLC28A3	S28A3_HUMAN	no	no
SLFN13	SLN13_HUMAN	no	no
SLFN12L	SN12L_HUMAN	no	no
SYNC	SYNCI_HUMAN	yes	yes
SYT1	SYT1_HUMAN	yes	yes
TRAFD1	TRAD1_HUMAN	yes	no
U6	SNR27_HUMAN	yes	yes
WDR85	DPH7_HUMAN	no	no

**Table 4:** Characteristics of patients.

		Initial Cohort (*n* = 832)		Survivors after 7 Days (*n* = 698)	
	All Cohort	Dead before Day 7	Alive at Day 7	Dead before Day 28	Alive at Day 28
	*n* = 832	*n* = 134	*n* = 698	*n* = 111	*n* = 587
Age (year)	67.3 ± 22.7 ^a^	70.8 ± 13.1	65.8 ± 24.0	71.4 ± 14.8	63.4 ± 24.5
Male sex (%)	467 (56.1)	78 (58.2)	389 (55.7)	66 (59.5)	323 (55)
Drotrecogin alpha (%)	407 (48.9)	59 (44)	348 (49.9)	44 (39.6)	304 (51.8)
Prior and preexisting conditions (%)					
Hypertension	297 (35.7)	53 (39.6)	244 (35.0)	43 (38.7)	201 (34.2)
Myocardial infarction	129 (15.5)	30 (22.4)	99 (14.2)	28 (25.2)	71 (12.1)
Congestive cardiomyopathy	67 (8.1)	7 (5.2)	60 (8.6)	20 (18)	40 (6.8)
Diabetes	169 (20.3)	30 (22.4)	139 (19.9)	27 (24.3)	112 (19.1)
Pancreatitis	30 (3.6)	5 (3.7)	25 (3.6)	4 (3.6)	21 (3.6)
Liver disease	14 (1.7)	3 (2.2)	11 (1.6)	4 (3.6)	7 (1.2)
COPD ^b^	226 (27.2)	39 (29.1)	187 (26.8)	36 (32.4)	151 (25.7)
Cancer	169 (20.3)	33 (24.6)	136 (19.5)	29 (26.1)	107 (18.2)
Apache II score	25 ± 10	28 ± 11.8	25 ± 10	28 ± 9	24 ± 10
SOFA score ^c^	8 ± 3	9.5 ± 3	8 ± 3	8 ± 3	8 ± 3
log(IL-6) ^d^	6.4 ± 3.1	7.4 ± 4.0	6.1 ± 2.9	6.3 ± 2.6	6.1 ± 2.9

^a^ Values are median ± inter-quartile interval. ^b^ COPD denotes chronic obstructive pulmonary disease, and APACHE II Acute Physiology and Chronic Health Evaluation II. ^c^ The organ components of the Sequential Organ Failure Assessment (SOFA) scores were provided by the PROWESS dataset. We calculated the sum of the organ component SOFA scores except for the neuronal ones (not included in the database). ^d^ IL-6 plasma levels measured at day 1 were expressed in logarithm base 10 due to scattered values. Due to rounding, not all percentages gave a total of 100.

**Table 5:** Significant covariates for GWAS.

	Survival at Day 7		Survival between Day 7 and Day 28	
	*p*-Value	OR (CI) ^a^	*p*-Value	OR (CI)
Age (years)	4.98 × 10^−4^	1.03 (1.01; 1.05)	1.62 × 10^−3^	1.03 (1.01; 1.05)
Gender (M/F)	NS	NS	NS	NS
Hypertension	NS	NS	NS	NS
Myocardial infarct	NS	NS	NS	NS
Cardiomyopathy	NS	NS	1.53 × 10^−3^	3.11 (1.52; 6.24)
Chronic obstructive pulmonary disease (COPD)	NS	NS	NS	NS
Diabetes	NS	NS	NS	NS
Liver disease	NS	NS	NS	NS
Malignancy	NS	NS	NS	NS
Pre-infusion APACHE score	NS	NS	NS	NS
Log of baseline IL-6 concentration	2.64 × 10^−6^	1.29 (1.16; 1.44)	NS	NS
Treatment by Activated Prot C or not	NS	NS	2.22 × 10^−2^	0.56 (0.33; 0.91)
baseline SOFA score (without neuro component)	4.77 × 10^−2^	1.10 (1.00; 1.22)	1.74 × 10^−2^	1.14 (1.02; 1.27)

^a^ Confidence Interval.

## Data Availability

Data is contained within the article or Supplementary Materials. Basic data is available on request.

## Supplementary Materials

The following are available online at www.mdpi.com/xxx/s1s.

## Abbreviations

*CISH*	cytokine inducible SH2 containing protein
eQTL	expression quantitative trait loci
FDR	false discovery rate
GWAS	genome-wide association studies
KEGG	Kyoto encyclopedia of genes and genomes
LPS	lipopolysaccharide
PTP	protein tyrosine phosphatase
RCTs	randomized clinical trials
ROC	receiving operating characteristic
RSAT	regulatory sequence analysis tools
SNP	single nucleotide polymorphism
SOFA	sequential organ failure assessment
SOCS	suppressor of cytokine signaling
