# Supplementary material for: Genetic Predisposition to the Mortality in Septic Shock Patients: From GWAS to the Identification of a Regulatory Variant Modulating the Activity of a CISH Enhancer
Source: Int J Mol Sci. 2021 May 29;22(11):5852. doi: 10.3390/ijms22115852 (PMC8198806; doi:10.3390/ijms22115852)
Supplement: Supplementary file 1 [file ijms-22-05852-s001.zip › ijms-1214840 suppl/Supplementary files/Supplementary_Table_1.pdf]

Supplementary Table 1. SNPs associated with early mortality with an FDR of 5% level

SNP	CHR:position	Alleles (MAF)	MAF (CEU/EUR ref panels)	Risk allele	Pc1df (qvalue)	effB (se_effB)	DL region	Genes containing SNP	Genes in LD
rs16840396	1 :240737932	C > T (0.013)	0.02/0.01	T	3.85E-07 (1.71E-02)	3,10 (0,61)	1:240723463-240737932	GREM2	
rs34737153	2 :134020386	G > A (0.019)	0.03/0.02	A	5.22E-07 (2.11E-02)	2,82 (0,56)	2:134020386-134022517	NCKAP5	
rs16857698	3 :145685067	A > G (0.014)	0.02/0.02	G	1.75E-09 (7.53E-04)	3,36 (0,56)	3:145665563-145685067		
rs5029231	3 :145701146	C > T (0.019)	0.02/0.02	T	1.37E-08 (1.73E-03)	2,97 (0,52)	3:145686379-145759412		
rs6763296	3 :145709314	T > C (0.018)	0.02/0.02	C	2.55E-09 (7.53E-04)	3,15 (0,53)	3:145686379-145759412		
rs16857836	3 :145752473	G > T (0.014)	0.02/0.02	T	5.51E-10 (4.89E-04)	3,50 (0,56)	3:145686379-145759412		
rs11948550	5 :14883436	G > A (0.074)	0.08/0.09	A	7.38E-07 (2.62E-02)	1,89 (0,38)	5:14880408-14883436		ANKH
rs17169594	7 :138101920	C > T (0.013)	0.01/0.01	T	1.44E-06 (4.12E-02)	3,14 (0,65)	7:138093347-138101920		
rs4544	8 :143994806	T > C (0.010)	0/0.00	C	8.86E-09 (1.31E-03)	4,40 (0,76)	8:143983592-144018027	CYP11B2	GML
rs11991278	8 :144001245	C > T (0.010)	0/0.01	T	8.48E-09 (1.31E-03)	4,40 (0,76)	8:143983592-144018027	CYP11B2	GML
rs6981918	8 :144007939	C > A (0.010)	0/0.01	A	8.74E-09 (1.31E-03)	4,39 (0,76)	8:143983592-144018027	CYP11B2	GML
rs956727	9 :86846933	A > G (0.009)	0.01/0.01	G	3.22E-08 (2.60E-03)	4,26 (0,77)	9:86814655-86862104	SLC28A3	
rs10867037	9 :140536098	C > T (0.036)	0.05/0.02	T	6.56E-07 (2.50E-02)	2,34 (0,47)	9:140455663-140536098	EHMT1	WDR85
rs11137198	9 :140638534	G > A (0.023)	0.05/0.02	A	1.21E-07 (8.25E-03)	2,71 (0,51)	9 :140638534-140638534	EHMT1	
rs12268257	10 :25864681	C > T (0.135)	0.14/0.15	T	1.31E-06 (3.88E-02)	1,66 (0,34)	10:25864681-25864681	GPR158	
rs16928895	10 :28734655	C > T (0.144)	0.17/0.14	T	1.27E-06 (3.88E-02)	1,64 (0,34)	10:28734655-28734655		
rs1362036	12 :112812282	C > T (0.017)	0.01/0.01	T	1.72E-06 (4.77E-02)	2,80 (0,58)	12:112568643-112889684	C12orf51	TRAFD1, RPL6, PTPN11
rs11066321	12 :112909396	T > C (0.015)	0.01/0.01	C	3.35E-07 (1.62E-02)	3,02 (0,59)	12:112819245-112985734	PTPN11	RPH3A, C12orf51
rs9668774	12 :112910267	G > A (0.015)	0.01/0.01	A	3.37E-07 (1.62E-02)	3,01 (0,59)	12:112819245-112985734	PTPN11	RPH3A, C12orf51
rs12301915	12 :112919869	C > A (0.015)	0.01/0.01	A	3.47E-07 (1.62E-02)	3,01 (0,59)	12:112819245-112985734	PTPN11	RPH3A, C12orf51
rs7974468	12 :112927208	G > A (0.013)	0.01/0.01	A	1.60E-08 (1.78E-03)	3,42 (0,60)	12:112819245-112985734	PTPN11	RPH3A, C12orf51
rs7975439	12 :112927272	C > T (0.015)	0.01/0.01	T	3.37E-07 (1.62E-02)	3,01 (0,59)	12:112819245-112985734	PTPN11	RPH3A, C12orf51
rs10849640	12 :119712137	G > A (0.116)	0.15/0.13	A	3.22E-08 (2.60E-03)	1,80 (0,32)	12:119712137-119725314		
rs10849641	12 :119721354	C > T (0.115)	0.15/0.13	T	2.65E-08 (2.60E-03)	1,80 (0,32)	12:119712137-119725314		
rs10849642	12 :119725314	C > T (0.117)	0.15/0.13	T	4.04E-08 (2.99E-03)	1,79 (0,32)	12:119712137-119725314		
rs2061815	16 :7070643	G > A (0.018)	0.01/0.03	A	4.09E-07 (1.73E-02)	2,84 (0,56)	16:7056818-7082344	RBFOX1	
rs9891869	17 :54516704	C > T (0.017)	0.03/0.01	T	3.30E-07 (1.62E-02)	2,89 (0,56)	17:54324640-54570315	ANKFN1	
rs9901040	17 :54534936	G > A (0.020)	0.03/0.01	A	1.30E-06 (3.88E-02)	2,63 (0,54)	17:54534936-54534936	ANKFN1	
rs7219938	17 :54548008	C > T (0.020)	0.03/0.01	T	1.30E-06 (3.88E-02)	2,63 (0,54)	17:54544766-54570315	ANKFN1	
rs7223494	17 :54552209	A > G (0.020)	0.03/0.01	G	6.76E-07 (2.50E-02)	2,70 (0,54)	17:54441957-54570315	ANKFN1	
rs9912206	17 :54557230	T > C (0.019)	0.03/0.01	C	3.40E-07 (1.62E-02)	2,78 (0,54)	17:54441957-54570315	ANKFN1	
rs2838103	21 :43147374	T > C (0.256)	0.25/0.26	C	1.24E-06 (3.88E-02)	1,52 (0,31)	21:43147374-43147374		
