# Supplementary material for: Genetic Predisposition to the Mortality in Septic Shock Patients: From GWAS to the Identification of a Regulatory Variant Modulating the Activity of a CISH Enhancer
Source: Int J Mol Sci. 2021 May 29;22(11):5852. doi: 10.3390/ijms22115852 (PMC8198806; doi:10.3390/ijms22115852)
Supplement: Supplementary file 1 [file ijms-22-05852-s001.zip › ijms-1214840 suppl/Supplementary files/Supplementary_Table_2.pdf]

Supplementary Table 2. SNPs associated with late mortality with an FDR of 5% level

SNP	CHR:position	Alleles (MAF)	MAF (CEU/EUR ref panels)	Risk allele	Pc1df (qvalue)	effB (se_effB)	DL region	Genes containing SNP	Genes in LD
rs359952	1 : 33187046	T > C (0.040)	0.02/0.03	C	1.23E-07 (3.24E-03)	2.36 (0.44)	1:33167504-33198782		SYNC
rs17442970	1 : 36887813	C > A (0.016)	0.02/0.02	A	2.36E-06 (2.93E-02)	3.18 (0.66)	1:36874213-36916787	OSCP1	
rs7526691	1 : 97736657	T > C (0.016)	0.01/0.02	C	5.15E-06 (4.31E-02)	2.77 (0.60)	1:97726100-97754565	DPYD-AS1. DPYD	
rs6692946	1 : 97748929	A > G (0.016)	0.01/0.02	G	2.66E-06 (3.18E-02)	2.87 (0.60)	1:97726100-97754565	DPYD-AS1. DPYD	
rs1509380	2 : 22717090	C > T (0.047)	0.05/0.05	T	9.68E-07 (1.58E-02)	2.12 (0.43)	2:22623946-22747258		
rs1107312	3 : 50539219	A > G (0.013)	0.01/0.01	G	4.55E-06 (3.88E-02)	3.36 (0.72)	3:50534635-50569775	CACNA2D2	
rs12491812	3 : 50556581	C > T (0.011)	0.01/0.01	T	4.18E-11 (1.25E-05)	5.10 (0.76)	3:50534635-50645413	CACNA2D2	C3orf18. HEMK1. CISH
rs12487817	3 : 50562566	T > C (0.011)	0.01/0.01	C	3.44E-06 (3.28E-02)	3.76 (0.80)	3:50534635-50645413		CACNA2D2. C3orf18. HEMK1. CISH
rs4688713	3 : 50566497	T > C (0.011)	0.01/0.01	C	3.27E-06 (3.28E-02)	3.77 (0.80)	3:50534635-50645413		CACNA2D2. C3orf18. HEMK1. CISH
rs2239753	3 : 50645158	T > C (0.011)	0.01/0.01	C	2.80E-11 (1.25E-05)	5.14 (0.76)	3:50555933-50645413	CISH	C3orf18. HEMK1. CACNA2D2
rs2239752	3 : 50645413	C > T (0.011)	0.01/0.01	T	5.43E-10 (4.86E-05)	4.59 (0.73)	3:50555933-50645413	CISH	C3orf18. HEMK1. CACNA2D2
rs2239751	3 : 50647888	A > C (0.011)	0.01/0.01	C	5.21E-10 (4.86E-05)	4.60 (0.73)	3:50531386-50875635	CISH	C3orf18. HEMK1. CACNA2D2. MAPKAPK3. DOCK3
rs743753	3 : 50651395	C > T (0.011)	0.01/0.01	T	5.21E-10 (4.86E-05)	4.60 (0.73)	3:50531386-50875635	MAPKAPK3	C3orf18. HEMK1. CACNA2D2. CISH. DOCK3
rs616689	3 : 50668532	G > A (0.014)	0.01/0.01	A	1.87E-10 (3.35E-05)	4.40 (0.68)	3:50647343-50751643	MAPKAPK3	CISH. DOCK3
rs9879397	3 : 50685642	G > A (0.012)	0.01/0.01	A	8.79E-09 (6.57E-04)	4.27 (0.73)	3:50647343-50751643	MAPKAPK3	CISH. DOCK3
rs2170840	3 : 50686517	A > C (0.014)	0.01/0.01	C	1.87E-10 (3.35E-05)	4.40 (0.68)	3:50647343-50751643	MAPKAPK3	CISH. DOCK3
rs12492982	3 : 50698155	C > T (0.011)	0.01/0.01	T	4.18E-11 (1.25E-05)	5.10 (0.76)	3:50531386-50875635	MAPKAPK3	C3orf18. HEMK1. CACNA2D2. CISH. DOCK3
rs2035484	3 : 50721892	A > G (0.011)	0.01/0.01	G	5.21E-10 (4.86E-05)	4.60 (0.73)	3:50721892-50721892	DOCK3	
rs17051403	3 : 50751643	C > A (0.011)	0.01/0.01	A	5.21E-10 (4.86E-05)	4.60 (0.73)	3:50531386-50875635	DOCK3	C3orf18. HEMK1. CACNA2D2. CISH. MAPKAPK3
rs17072628	3 : 65229760	G > A (0.012)	0.01/0.01	A	8.25E-09 (6.57E-04)	4.12 (0.70)	3:65214495-65241577		
rs9856368	3 : 187756856	G > T (0.067)	0.08/0.08	T	6.84E-07 (1.20E-02)	1.97 (0.39)	3:187756856-187759204		
rs6852672	4 : 125147414	C > A (0.220)	0.15/0.18	A	3.49E-06 (3.28E-02)	1.52 (0.32)	4:125054040-125182624		
rs12654328	5 : 65085672	A > G (0.029)	0.02/0.01	G	6.42E-07 (1.15E-02)	2.51 (0.50)	5:65033368-65124990	NLN	
rs6449969	5 : 67903602	G > A (0.044)	0.04/0.05	A	1.92E-07 (3.74E-03)	2.30 (0.43)	5:67890719-67924890		

rs7726677	5 : 67913510	A > G (0.040)	0.04/0.05	G	1.85E-07 (3.69E-03)	2.36 (0.44)	5:67890719-67933054		
rs16897944	5 : 67915481	A > G (0.044)	0.04/0.05	G	1.98E-07 (3.78E-03)	2.30 (0.43)	5:67890719-67933054		
rs3797817	5 : 108284342	A > G (0.020)	0.02/0.02	G	7.59E-07 (1.28E-02)	2.77 (0.55)	5:108262268-108378678	FER	
rs6910170	6 : 121377016	T > C (0.023)	0.04/0.03	C	2.13E-06 (2.72E-02)	2.69 (0.56)	6:121377016-121465569	C6orf170	
rs11987625	8 : 20521138	G > A (0.011)	0/0.01	A	4.23E-06 (3.71E-02)	3.27 (0.70)	8:20521138-20521138		
rs11994554	8 : 66228144	G > A (0.040)	0.07/0.05	A	1.38E-06 (1.96E-02)	2.20 (0.45)	8:66197574-66265924		
rs2175946	8 : 66240339	T > C (0.041)	0.07/0.05	C	3.19E-06 (3.28E-02)	2.14 (0.45)	8:66197574-66265924		
rs7828588	8 : 66247105	C > T (0.040)	0.07/0.05	T	2.11E-06 (2.72E-02)	2.17 (0.45)	8:66197574-66265924		
rs1514524	8 : 66321181	G > A (0.142)	0.16/0.12	A	1.60E-06 (2.17E-02)	1.62 (0.33)	8:66321181-66335092		
rs7840669	8 : 89929277	A > G (0.015)	0.01/0.01	G	2.38E-08 (1.53E-03)	3.58 (0.63)	8:89901960-90133835		
rs3005838	9 : 38654385	A > G (0.033)	0.03/0.03	G	9.02E-08 (2.61E-03)	2.53 (0.47)	9:38645206-38665726		U6. FAM201A
rs7096890	10 : 91141497	T > C (0.014)	0.01/0.03	C	1.17E-06 (1.79E-02)	3.34 (0.68)	10:91141497-91150689	IFIT1B	LIPA
rs10887952	10 : 91145054	C > T (0.013)	0.01/0.03	T	1.17E-06 (1.79E-02)	3.34 (0.68)	10:91141497-91150689	LIPA	IFIT1B
rs4575240	11 : 37995616	G > A (0.009)	0/0.01	A	4.46E-06 (3.84E-02)	3.46 (0.74)	11:37843754-38006395		
rs7300564	12 : 79594953	C > T (0.014)	0.01/0.02	T	2.48E-06 (3.04E-02)	3.17 (0.66)	12:79587426-79600641	SYT1	
rs10778678	12 : 79944776	A > G (0.025)	0.01/0.01	G	7.24E-08 (2.24E-03)	2.81 (0.51)	12:79919466-80080618		PAWR
rs10861992	12 : 79946035	C > G (0.026)	0.01/0.01	G	1.66E-07 (3.55E-03)	2.72 (0.51)	12:79919466-80080618		PAWR
rs10861993	12 : 79946103	G > A (0.027)	0.01/0.01	A	1.72E-07 (3.59E-03)	2.72 (0.51)	12:79919466-80080618		PAWR
rs12230772	12 : 79962814	A > G (0.027)	0.01/0.01	G	1.66E-07 (3.55E-03)	2.72 (0.51)	12:79919466-80080618		PAWR
rs11114185	12 : 79970339	A > G (0.025)	0.01/0.01	G	7.24E-08 (2.24E-03)	2.81 (0.51)	12:79919466-80080618	PAWR	
rs7953683	12 : 79993704	C > T (0.024)	0.01/0.01	T	3.07E-08 (1.72E-03)	2.90 (0.52)	12:79919466-80080618	PAWR	
rs7955388	12 : 80006683	G > A (0.025)	0.01/0.01	A	7.24E-08 (2.24E-03)	2.81 (0.51)	12:79919466-80080618	PAWR	
rs8176875	12 : 80014188	T > C (0.024)	0.01/0.01	C	7.24E-08 (2.24E-03)	2.81 (0.51)	12:79919466-80080618	PAWR	
rs7305134	12 : 80015559	G > T (0.025)	0.01/0.01	T	7.24E-08 (2.24E-03)	2.81 (0.51)	12:79919466-80080618	PAWR	
rs7294911	12 : 80023110	G > A (0.025)	0.01/0.01	A	7.53E-08 (2.25E-03)	2.81 (0.51)	12:79919466-80080618	PAWR	
rs9651966	12 : 80028738	C > T (0.027)	0.01/0.01	T	1.66E-07 (3.55E-03)	2.72 (0.51)	12:79919466-80080618	PAWR	
rs2049589	12 : 80029516	T > G (0.025)	0.01/0.01	G	7.24E-08 (2.24E-03)	2.81 (0.51)	12:79919466-80080618	PAWR	
rs4441080	12 : 80030707	T > C (0.025)	0.01/0.01	C	7.24E-08 (2.24E-03)	2.81 (0.51)	12:79919466-80080618	PAWR	
rs10862006	12 : 80041539	A > G (0.019)	0.01/0.01	G	1.28E-07 (3.28E-03)	2.99 (0.56)	12:79939349-80059229	PAWR	
rs2049590	12 : 80043160	C > A (0.026)	0.01/0.01	A	1.78E-07 (3.63E-03)	2.72 (0.51)	12:79919466-80113350	PAWR	
rs10862007	12 : 80049965	A > G (0.024)	0.01/0.01	G	7.24E-08 (2.24E-03)	2.81 (0.51)	12:79919466-80113350	PAWR	
rs7134813	12 : 80055232	A > G (0.026)	0.01/0.01	G	6.84E-08 (2.24E-03)	2.82 (0.51)	12:79919466-80113350	PAWR	
rs1880883	12 : 80055766	A > G (0.027)	0.01/0.01	G	1.66E-07 (3.55E-03)	2.72 (0.51)	12:79919466-80113350	PAWR	

rs7300145	12 : 80062156	T > C (0.027)	0.01/0.01	C	1.66E-07 (3.55E-03)	2.72 (0.51)	12:79919466-80113350	PAW R	
rs10778686	12 : 80071955	G > A (0.025)	0.01/0.01	A	7.24E-08 (2.24E-03)	2.81 (0.51)	12:79919466-80113350	PAWR	
rs7307647	12 : 80076418	T > C (0.025)	0.01/0.01	C	7.24E-08 (2.24E-03)	2.81 (0.51)	12:79919466-80113350	PAWR	
rs2430866	12 : 80080618	T > C (0.025)	0.01/0.01	C	7.24E-08 (2.24E-03)	2.81 (0.51)	12:79919466-80113350	PAWR	
rs2694831	12 : 80086780	G > A (0.027)	0.01/0.01	A	1.66E-07 (3.55E-03)	2.72 (0.51)	12:79919466-80113350		PAWR
rs6539471	12 : 80089395	T > C (0.026)	0.01/0.01	C	6.17E-08 (2.24E-03)	2.82 (0.51)	12:79919466-80113350		PAWR
rs11114218	12 : 80090750	T > C (0.027)	0.01/0.01	C	1.66E-07 (3.55E-03)	2.72 (0.51)	12:79919466-80113350		PAWR
rs711159	12 : 80108416	C > T (0.024)	0.01/0.01	T	1.31E-06 (1.92E-02)	2.70 (0.55)	12:80028738-80113350		PAWR
rs1882182	12 : 81723387	G > A (0.069)	0.11/0.09	A	1.47E-06 (2.02E-02)	1.91 (0.39)	12:81706869-81827652	PPFIA2	
rs10862295	12 : 81727898	A > G (0.070)	0.11/0.09	G	2.01E-06 (2.64E-02)	1.89 (0.39)	12:81706869-81827652	PPFIA2	
rs2060206	12 : 81746835	C > T (0.068)	0.11/0.09	T	1.47E-06 (2.02E-02)	1.91 (0.39)	12:81706869-81827652	PPFIA2	
rs527603	13 : 30691576	T > C (0.014)	0.01/0.01	C	7.23E-07 (1.25E-02)	3.23 (0.64)	13:30691471-30716111		
rs661049	13 : 30696259	C > T (0.015)	0.01/0.01	T	2.17E-06 (2.74E-02)	3.05 (0.63)	13:30691471-30716111		
rs7992136	13 : 77110530	A > C (0.021)	0.03/0.02	C	1.33E-06 (1.92E-02)	2.69 (0.55)	13:77097654-77127812		
rs7317445	13 : 77127812	T > C (0.021)	0.03/0.02	C	2.58E-06 (3.12E-02)	2.61 (0.54)	13:77097654-77127812		
rs4646220	13 : 99374223	G > A (0.016)	0.01/0.01	A	2.87E-06 (3.28E-02)	2.86 (0.60)	13:99373095-99374223	SLC15A1	
rs1756650	14 : 87741025	A > G (0.074)	0.11/0.10	G	2.91E-06 (3.28E-02)	1.83 (0.39)	14:87741025-87745006		
rs943154	14 : 87744545	A > C (0.075)	0.11/0.10	C	5.14E-06 (4.31E-02)	1.80 (0.39)	14:87741025-87745006		
rs7178141	15 : 72726168	C > T (0.049)	0.05/0.06	T	3.62E-06 (3.28E-02)	2.03 (0.43)	15:72718691-72875842		ARIH1
rs2899780	15 : 72738115	G > A (0.049)	0.05/0.06	A	3.62E-06 (3.28E-02)	2.03 (0.43)	15:72718691-72875842		ARIH1
rs2415137	15 : 72738293	A > G (0.049)	0.05/0.06	G	3.77E-06 (3.38E-02)	2.03 (0.43)	15:72718691-72875842		ARIH1
rs8026681	15 : 72784482	C > T (0.049)	0.05/0.06	T	3.62E-06 (3.28E-02)	2.03 (0.43)	15:72718691-72875842	ARIH1	
rs8038734	15 : 72811402	A > G (0.049)	0.05/0.06	G	3.62E-06 (3.28E-02)	2.03 (0.43)	15:72718691-72875842	ARIH1	
rs10438370	15 : 72822876	G > T (0.049)	0.05/0.06	T	3.62E-06 (3.28E-02)	2.03 (0.43)	15:72718691-72875842	ARIH1	
rs7177472	15 : 72843785	G > T (0.049)	0.05/0.06	T	3.62E-06 (3.28E-02)	2.03 (0.43)	15:72718691-72923709	ARIH1	
rs4776602	15 : 72846354	A > G (0.049)	0.05/0.06	G	3.62E-06 (3.28E-02)	2.03 (0.43)	15:72718691-72923709	ARIH1	
rs8037282	15 : 72873727	C > T (0.049)	0.05/0.06	T	3.62E-06 (3.28E-02)	2.03 (0.43)	15:72718691-72923709	ARIH1	
rs4777517	15 : 72875842	G > A (0.049)	0.05/0.06	A	3.62E-06 (3.28E-02)	2.03 (0.43)	15:72718691-72923709	ARIH1	
rs7216725	17 : 29273786	C > T (0.014)	0.01/0.01	T	3.26E-06 (3.28E-02)	3.01 (0.63)	17:29075831-29285424	ADAP2	CRLF3. ATAD5
rs16968287	17 : 29275101	G > A (0.014)	0.01/0.01	A	3.18E-06 (3.28E-02)	3.01 (0.63)	17:29075831-29285424	ADAP2	CRLF3. ATAD5
rs8067480	17 : 29278968	T > C (0.014)	0.01/0.01	C	3.18E-06 (3.28E-02)	3.01 (0.63)	17:29075831-29285424	ADAP2	CRLF3. ATAD5
rs8076569	17 : 29280037	T > C (0.014)	0.01/0.01	C	3.18E-06 (3.28E-02)	3.01 (0.63)	17:29075831-29285424	ADAP2	CRLF3. ATAD5
rs8066682	17 : 29288195	G > A (0.014)	0.01/0.01	A	3.07E-06 (3.28E-02)	3.01 (0.63)	17:29285901-29323511	RNF135	

rs9675277	17 : 29290783	C > T (0.014)	0.01/0.01	T	3.07E-06 (3.28E-02)	3.01 (0.63)	17:29285901-29323511		RNF135
rs7219775	17 : 29297595	G > A (0.013)	0.01/0.02	A	1.11E-06 (1.78E-02)	3.18 (0.64)	17:29285901-29323511	RNF135	
rs7221473	17 : 29297910	C > G (0.014)	0.01/0.01	G	3.07E-06 (3.28E-02)	3.01 (0.63)	17:29285901-29323511	RNF135	
rs7220071	17 : 29304366	C > T (0.014)	0.01/0.01	T	3.07E-06 (3.28E-02)	3.01 (0.63)	17:29285901-29323511	RNF135	
rs9915124	17 : 29308489	G > T (0.013)	0.01/0.01	T	1.21E-06 (1.81E-02)	3.17 (0.64)	17:29285901-29323511	RNF135	
rs7221226	17 : 29312871	T > C (0.013)	0.01/0.01	C	1.18E-06 (1.79E-02)	3.18 (0.64)	17:29285901-29323511	RNF135	
rs2340518	17 : 29313078	A > G (0.013)	0.01/0.01	G	9.31E-07 (1.55E-02)	3.20 (0.64)	17:29285901-29323511	RNF135	
rs8074481	17 : 29330544	C > T (0.014)	0.01/0.02	T	3.14E-06 (3.28E-02)	3.01 (0.63)	17:29317076-29334058		RNF135
rs1434590	17 : 31939956	G > A (0.009)	0.005/0.00	A	4.39E-06 (3.82E-02)	3.72 (0.80)	17:31926894-31987375	ASIC2	
rs7214197	17 : 33777798	G > A (0.014)	0.01/0.00	A	1.10E-07 (2.99E-03)	3.43 (0.63)	17:33775513-33806841		SLFN13. SLFN12L
rs7215341	17 : 51520986	C > A (0.030)	0.01/0.03	A	9.61E-08 (2.69E-03)	2.52 (0.46)	17:51519876-51590268		
rs1502522	17 : 51544776	A > G (0.029)	0.01/0.03	G	2.57E-08 (1.53E-03)	2.64 (0.47)	17:51519876-51590268		
rs1393467	17 : 51560869	T > C (0.029)	0.01/0.03	C	2.57E-08 (1.53E-03)	2.64 (0.47)	17:51519876-51590268		
rs1036840	18 : 67217542	C > T (0.041)	0.04/0.06	T	4.22E-06 (3.71E-02)	2.10 (0.45)	18:67195941-67242127		
rs4381690	18 : 67250513	G > A (0.037)	0.02/0.05	A	5.75E-07 (1.05E-02)	2.32 (0.46)	18:67250305-67271649		
rs17271418	19 : 47985220	G > A (0.073)	0.12/0.08	A	2.00E-06 (2.64E-02)	1.88 (0.39)	19:47985220-47990889	KPTN	NAPA
rs2232619	20 : 37002589	G > A (0.013)	0.02/0.01	A	5.42E-06 (4.50E-02)	3.09 (0.67)	20:37002589-37002589	LBP	
