# Supplementary material for: Genetic Predisposition to the Mortality in Septic Shock Patients: From GWAS to the Identification of a Regulatory Variant Modulating the Activity of a CISH Enhancer
Source: Int J Mol Sci. 2021 May 29;22(11):5852. doi: 10.3390/ijms22115852 (PMC8198806; doi:10.3390/ijms22115852)
Supplement: Supplementary file 1 [file ijms-22-05852-s001.zip › ijms-1214840 suppl/Supplementary files/Supplementary_Table_3.pdf]

Supplementary Table 3. List of proteins encoded by genes associated with sepsis and their direct interactors

HGNC symbol	UniProt/SwissProt ID
YWHAB	1433B_HUMAN
YWHAZ	1433Z_HUMAN
EIF4EBP1	4EBP1_HUMAN
ABI1	ABI1_HUMAN
ABL1	ABL1_HUMAN
ACTC1	ACTC_HUMAN
ADAP2	ADAP2_HUMAN
AHSA1	AHSA1_HUMAN
ANKH	ANKH_HUMAN
APC	APC_HUMAN
ARIH1	ARI1_HUMAN
ASIC2	ASIC2_HUMAN
ATAD5	ATAD5_HUMAN
ATM	ATM_HUMAN
ATN1	ATN1_HUMAN
ATP5C1	ATPG_HUMAN
ATXN1	ATX1_HUMAN
ATXN2	ATX2_HUMAN
BAG6	BAG6_HUMAN
BCR	BCR_HUMAN
BST1	BST1_HUMAN
C4BPA	C4BPA_HUMAN
CACNA1A	CAC1A_HUMAN
CACNB3	CACB3_HUMAN
CACNB4	CACB4_HUMAN
CDH1	CADH1_HUMAN
CALM3	CALM_HUMAN
CALM2	CALM_HUMAN
CALM1	CALM_HUMAN
NEDD9	CASL_HUMAN
CBL	CBL_HUMAN
CBX3	CBX3_HUMAN
CD14	CD14_HUMAN
CD33	CD33_HUMAN
CDC23	CDC23_HUMAN
CDC37	CDC37_HUMAN
CDC5L	CDC5L_HUMAN
CEACAM1	CEAM1_HUMAN
CELF3	CELF3_HUMAN
ITGB3BP	CENPR_HUMAN
CENPT	CENPT_HUMAN
CHN1	CHIN_HUMAN
CISH	CISH_HUMAN
CPLX1	CPLX1_HUMAN
CRKL	CRKL_HUMAN
CRK	CRK_HUMAN
CRLF3	CRLF3_HUMAN
CSF3R	CSF3R_HUMAN
CSNK2A1	CSK21_HUMAN
COPS4	CSN4_HUMAN
COPS6	CSN6_HUMAN
CTBP1	CTBP1_HUMAN
CTBP2	CTBP2_HUMAN
CTNNB1	CTNB1_HUMAN
CTNND1	CTND1_HUMAN
CTNNAL1	CTNL1_HUMAN
CXCR4	CXCR4_HUMAN
DAPK3	DAPK3_HUMAN
DDR1	DDR1_HUMAN
DDX3X	DDX3X_HUMAN
DDX58	DDX58_HUMAN
DES	DESM_HUMAN
DMRTB1	DMRTB_HUMAN
DOCK3	DOCK3_HUMAN
DPYD	DPYD_HUMAN
DRD2	DRD2_HUMAN
DVL1	DVL1_HUMAN
DVL2	DVL2_HUMAN
DNM1	DYN1_HUMAN
DNM2	DYN2_HUMAN
DNM3	DYN3_HUMAN
EEF1G	EF1G_HUMAN
EGFR	EGFR_HUMAN
EHMT1	EHMT1_HUMAN
EHMT2	EHMT2_HUMAN
EPOR	EPOR_HUMAN
EPS15	EPS15_HUMAN
ERBB2	ERBB2_HUMAN
ERBB3	ERBB3_HUMAN
ERBB4	ERBB4_HUMAN
ERC2	ERC2_HUMAN
ETV5	ETV5_HUMAN
EZH2	EZH2_HUMAN
PTK2	FAK1_HUMAN
PTK2B	FAK2_HUMAN
FBL	FBRL_HUMAN
FCRL3	FCRL3_HUMAN
FER	FER_HUMAN
FGF1	FGF1_HUMAN
FGF2	FGF2_HUMAN
FLT3	FLT3_HUMAN
FTH1	FRIH_HUMAN
FRS2	FRS2_HUMAN
FRS3	FRS3_HUMAN
FYN	FYN_HUMAN
GAB1	GAB1_HUMAN
GAB2	GAB2_HUMAN
GAREM	GAREM_HUMAN
CCKBR	GASR_HUMAN
NR3C1	GCR_HUMAN
GHR	GHR_HUMAN
GIT1	GIT1_HUMAN
GNA12	GNA12_HUMAN
GOLGA2	GOGA2_HUMAN
GOLM1	GOLM1_HUMAN
GOPC	GOPC_HUMAN
GOSR1	GOSR1_HUMAN
GOSR2	GOSR2_HUMAN
GPR158	GP158_HUMAN
GCA	GRAN_HUMAN
GRB2	GRB2_HUMAN
GRB7	GRB7_HUMAN
GRIA2	GRIA2_HUMAN

GRIA3	GRIA3_HUMAN
HSPA5	GRP78_HUMAN
GLG1	GSLG1_HUMAN
GTF2I	GTF2I_HUMAN
HIST1H3H	H31_HUMAN
HIST1H3I	H31_HUMAN
HIST1H3J	H31_HUMAN
HIST1H3A	H31_HUMAN
HIST1H3B	H31_HUMAN
HIST1H3C	H31_HUMAN
HIST1H3D	H31_HUMAN
HIST1H3E	H31_HUMAN
HIST1H3F	H31_HUMAN
HIST1H3G	H31_HUMAN
HEMK1	HEMK1_HUMAN
HNRNPF	HNRPF_HUMAN
SYNCRIP	HNRPQ_HUMAN
RARRES3	HRSL4_HUMAN
HSP90AA1	HS90A_HUMAN
HSP90AB1	HS90B_HUMAN
HSFX2	HSFX1_HUMAN
HSFX1	HSFX1_HUMAN
HSPA6	HSP76_HUMAN
HSPB1	HSPB1_HUMAN
HSPB2-C11orf52	HSPB2_HUMAN
CAST	ICAL_HUMAN
EIF4E2	IF4E2_HUMAN
EIF6	IF6_HUMAN
IGF1R	IGF1R_HUMAN
NFKBIA	IKBA_HUMAN
NFKBIB	IKBB_HUMAN
CHUK	IKKA_HUMAN
IKBKB	IKKB_HUMAN
CSF2RB	IL3RB_HUMAN
IL4R	IL4RA_HUMAN
IL6ST	IL6RB_HUMAN
IFNAR1	INAR1_HUMAN
INSR	INSR_HUMAN
PKIG	IPKG_HUMAN
IRF6	IRF6_HUMAN
IRS1	IRS1_HUMAN
JAK1	JAK1_HUMAN
JAK2	JAK2_HUMAN
KIR2DL1	KI2L1_HUMAN
KIR2DL3	KI2L3_HUMAN
KIFAP3	KIFA3_HUMAN
KIT	KIT_HUMAN
PRKCA	KPCA_HUMAN
PRKCB	KPCB_HUMAN
PRKCI	KPCI_HUMAN
PRKCH	KPCL_HUMAN
PRKCQ	KPCT_HUMAN
PRKCZ	KPCZ_HUMAN
KPTN	KPTN_HUMAN
LBP	LBP_HUMAN
LEPR	LEPR_HUMAN
PPFIA1	LIPA1_HUMAN

PPFIA2	LIPA2_HUMAN
PPFIBP1	LIPB1_HUMAN
LAMTOR3	LTOR3_HUMAN
LXN	LXN_HUMAN
LZTR1	LZTR1_HUMAN
LZTS2	LZTS2_HUMAN
MAP3K4	M3K4_HUMAN
MAP3K8	M3K8_HUMAN
MAP4K4	M4K4_HUMAN
MAGED1	MAGD1_HUMAN
MAPKAPK3	MAPK3_HUMAN
MAX	MAX_HUMAN
MET	MET_HUMAN
MAPK3	MK03_HUMAN
MAPK11	MK11_HUMAN
MAPK14	MK14_HUMAN
CBFA2T2	MTG8R_HUMAN
MTTP	MTP_HUMAN
NCK1	NCK1_HUMAN
NCKAP5	NCKP5_HUMAN
NCOA1	NCOA1_HUMAN
NCOA2	NCOA2_HUMAN
IKBKG	NEMO_HUMAN
NFKB1	NFKB1_HUMAN
NFKB2	NFKB2_HUMAN
NGF	NGF_HUMAN
KLRC1	NKG2A_HUMAN
GRIN2B	NMDE2_HUMAN
NOC2L	NOC2L_HUMAN
NPAS2	NPAS2_HUMAN
IVNS1ABP	NS1BP_HUMAN
NSF	NSF_HUMAN
NTRK1	NTRK1_HUMAN
MT-ND1	NU1M_HUMAN
NUMBL	NUMBL_HUMAN
TP53	P53_HUMAN
PYCRL	P5CR3_HUMAN
PIK3R1	P85A_HUMAN
PIK3R2	P85B_HUMAN
PAWR	PAWR_HUMAN
PXN	PAXI_HUMAN
PCBP4	PCBP4_HUMAN
CBFB	PEBB_HUMAN
PDGFRA	PGFRA_HUMAN
PDGFRB	PGFRB_HUMAN
PCBD1	PHS_HUMAN
PICK1	PICK1_HUMAN
PILRB	PILRB_HUMAN
PITX2	PITX2_HUMAN
PIK3CG	PK3CG_HUMAN
PLEKHA5	PKHA5_HUMAN
PLD1	PLD1_HUMAN
PML	PML_HUMAN
PPARG	PPARG_HUMAN
LGALS14	PPL13_HUMAN
PRRC2A	PRC2A_HUMAN
PRDX4	PRDX4_HUMAN

PRNP	PRIO_HUMAN
PRLR	PRLR_HUMAN
PSMA3	PSA3_HUMAN
PSMF1	PSMF1_HUMAN
PSEN1	PSN1_HUMAN
PSEN2	PSN2_HUMAN
PTPN11	PTN11_HUMAN
PTPN1	PTN1_HUMAN
PTPN6	PTN6_HUMAN
PTPRF	PTPRF_HUMAN
PTPRG	PTPRG_HUMAN
PTPRS	PTPRS_HUMAN
QKI	QKI_HUMAN
RAB2A	RAB2A_HUMAN
RBM24	RBM24_HUMAN
RELB	RELB_HUMAN
REL	REL_HUMAN
RBFOX1	RFOX1_HUMAN
RBFOX2	RFOX2_HUMAN
RHOXF2	RHXF2_HUMAN
RING1	RING1_HUMAN
RPL30	RL30_HUMAN
RPL4	RL4_HUMAN
RPL6	RL6_HUMAN
RPL8	RL8_HUMAN
RNF135	RN135_HUMAN
ROS1	ROS1_HUMAN
RAPGEF1	RPGF1_HUMAN
RPIA	RPIA_HUMAN
RRAS2	RRAS2_HUMAN
RPS11	RS11_HUMAN
RPS13	RS13_HUMAN
RPS21	RS21_HUMAN
RPSA	RSSA_HUMAN
RTN4	RTN4_HUMAN
S100A13	S10AD_HUMAN
SEL1L	SE1L1_HUMAN
SETD1A	SET1A_HUMAN
INPP5D	SHIP1_HUMAN
SIRPA	SHPS1_HUMAN
SIGLEC12	SIG12_HUMAN
SIN3A	SIN3A_HUMAN
SIT1	SIT1_HUMAN
SLAMF1	SLAF1_HUMAN
SLK	SLK_HUMAN
SMAD2	SMAD2_HUMAN
SMAD3	SMAD3_HUMAN
NAPA	SNAA_HUMAN
NAPB	SNAB_HUMAN
SNAP23	SNP23_HUMAN
SNAP25	SNP25_HUMAN
SNRNP27	SNR27_HUMAN
SOCS3	SOCS3_HUMAN

SOS1	SOS1_HUMAN
SMS	SPSY_HUMAN
CTTN	SRC8_HUMAN
SRC	SRC_HUMAN
SRPK2	SRPK2_HUMAN
STAT5A	STA5A_HUMAN
STAT5B	STA5B_HUMAN
STAT1	STAT1_HUMAN
STAT3	STAT3_HUMAN
STOM	STOM_HUMAN
STON2	STON2_HUMAN
STX12	STX12_HUMAN
STX1A	STX1A_HUMAN
STX2	STX2_HUMAN
STX3	STX3_HUMAN
STX4	STX4_HUMAN
STX5	STX5_HUMAN
STX7	STX7_HUMAN
SUMO3	SUMO3_HUMAN
SYNC	SYNCI_HUMAN
SYT1	SYT1_HUMAN
TSC22D4	T22D4_HUMAN
TERT	TERT_HUMAN
RELA	TF65_HUMAN
TFCP2	TFCP2_HUMAN
TFDP1	TFDP1_HUMAN
TCF3	TFE2_HUMAN
TFPT	TFPT_HUMAN
THAP1	THAP1_HUMAN
TEK	TIE2_HUMAN
TMF1	TMF1_HUMAN
TNIP1	TNIP1_HUMAN
TNFRSF1A	TNR1A_HUMAN
MPL	TPOR_HUMAN
TRA2A	TRA2A_HUMAN
TRIM15	TRI15_HUMAN
TRIM32	TRI32_HUMAN
TRPC3	TRPC3_HUMAN
UBE2L3	UB2L3_HUMAN
UBE2L6	UB2L6_HUMAN
AXL	UFO_HUMAN
VAMP2	VAMP2_HUMAN
VAMP3	VAMP3_HUMAN
VAMP8	VAMP8_HUMAN
VAV2	VAV2_HUMAN
FLT1	VGFR1_HUMAN
KDR	VGFR2_HUMAN
FLT4	VGFR3_HUMAN
VPS25	VPS25_HUMAN
WT1	WT1_HUMAN
ZDHHC17	ZDH17_HUMAN
