# Supplementary material for: Genetic Predisposition to the Mortality in Septic Shock Patients: From GWAS to the Identification of a Regulatory Variant Modulating the Activity of a CISH Enhancer
Source: Int J Mol Sci. 2021 May 29;22(11):5852. doi: 10.3390/ijms22115852 (PMC8198806; doi:10.3390/ijms22115852)
Supplement: Supplementary file 1 [file ijms-22-05852-s001.zip › ijms-1214840 suppl/Supplementary files/Supplementary_Table_4.pdf]

Supplementary Table 4. KEGG and BIOCARTA pathways significantly enriched in genes of the sub-network of proteins encoded by associated genes and their direct interactors. Correction for multiple testing was performed by Benjamini-Hochberg correction at 5%.

Term[a]	Count[b]	LT[c]	B1[d]	B2[e]	P Value[f]	Benjamini[g]
hsa05200:Pathways in cancer	55	203	328	5085	8,18E-21	9,08E-19
hsa05220:Chronic myeloid leukemia	25	203	75	5085	2,50E-16	1,23E-14
hsa04722:Neurotrophin signaling pathway	29	203	124	5085	1,70E-14	6,28E-13
hsa04012:ErbB signaling pathway	23	203	87	5085	1,15E-12	3,19E-11
hsa04010:MAPK signaling pathway	39	203	267	5085	1,71E-12	3,80E-11
hsa05221:Acute myeloid leukemia	18	203	58	5085	3,67E-11	6,80E-10
hsa04660:T cell receptor signaling pathway	23	203	108	5085	1,18E-10	1,87E-09
hsa05215:Prostate cancer	21	203	89	5085	1,34E-10	1,86E-09
hsa04130:SNARE interactions in vesicular transport	14	203	38	5085	9,43E-10	1,16E-08
hsa04630:Jak-STAT signaling pathway	26	203	155	5085	1,15E-09	1,27E-08
hsa05212:Pancreatic cancer	18	203	72	5085	1,54E-09	1,55E-08
hsa04520:Adherens junction	18	203	77	5085	4,67E-09	4,32E-08
hsa05210:Colorectal cancer	18	203	84	5085	1,90E-08	1,62E-07
hsa04662:B cell receptor signaling pathway	17	203	75	5085	2,29E-08	1,82E-07
hsa04910:Insulin signaling pathway	22	203	135	5085	5,22E-08	3,86E-07
hsa04062:Chemokine signaling pathway	26	203	187	5085	5,94E-08	4,12E-07
hsa04920:Adipocytokine signaling pathway	15	203	67	5085	2,37E-07	1,55E-06
hsa04510:Focal adhesion	26	203	201	5085	2,51E-07	1,55E-06
hsa05214:Glioma	14	203	63	5085	7,56E-07	4,42E-06
hsa04210:Apoptosis	16	203	87	5085	1,21E-06	6,71E-06
h_metPathway:Signaling of Hepatocyte Growth Factor Receptor	14	124	34	1437	1,40E-06	2,89E-04
hsa04620:Toll-like receptor signaling pathway	17	203	101	5085	1,72E-06	9,07E-06
hsa05120:Epithelial cell signaling in Helicobacter pylori infection	14	203	68	5085	1,90E-06	9,59E-06
hsa04144:Endocytosis	23	203	184	5085	2,72E-06	1,31E-05
hsa05213:Endometrial cancer	12	203	52	5085	4,27E-06	1,98E-05

hsa04370:VEGF signaling pathway	14	203	75	5085	6,00E-06	2,66E-05
hsa04664:Fc epsilon RI signaling pathway	14	203	78	5085	9,40E-06	4,01E-05
h_TPOPathway:TPO Signaling Pathway	10	124	20	1437	1,39E-05	1,43E-03
hsa05218:Melanoma	13	203	71	5085	1,81E-05	7,43E-05
hsa04666:Fc gamma R-mediated phagocytosis	15	203	95	5085	1,85E-05	7,31E-05
h_egfPathway:EGF Signaling Pathway	11	124	26	1437	2,41E-05	1,65E-03
hsa04621:NOD-like receptor signaling pathway	12	203	62	5085	2,54E-05	9,71E-05
h_ghPathway:Growth Hormone Signaling Pathway	10	124	22	1437	3,54E-05	1,82E-03
hsa05223:Non-small cell lung cancer	11	203	54	5085	4,06E-05	1,50E-04
hsa04670:Leukocyte transendothelial migration	16	203	118	5085	5,57E-05	2,00E-04
hsa04650:Natural killer cell mediated cytotoxicity	17	203	133	5085	6,22E-05	2,16E-04
hsa04930:Type II diabetes mellitus	10	203	47	5085	7,55E-05	2,54E-04
h_nthiPathway:NFkB activation by Nontypeable Hemophilus influenzae	10	124	24	1437	8,02E-05	3,30E-03
hsa05211:Renal cell carcinoma	12	203	70	5085	8,17E-05	2,67E-04
h_cblPathway:CBL mediated ligand-induced downregulation of EGF receptors	7	124	11	1437	1,13E-04	3,87E-03
h_pdgfPathway:PDGF Signaling Pathway	10	124	25	1437	1,16E-04	3,42E-03
h_her2Pathway:Role of ERBB2 in Signal Transduction and Oncology	9	124	21	1437	1,81E-04	4,65E-03
h_cxcr4Pathway:CXCR4 Signaling Pathway	9	124	23	1437	3,76E-04	8,58E-03
h_epoPathway:EPO Signaling Pathway	8	124	18	1437	4,08E-04	8,36E-03
hsa05222:Small cell lung cancer	12	203	84	5085	4,30E-04	1,36E-03
hsa04622:RIG-I-like receptor signaling pathway	11	203	71	5085	4,34E-04	1,34E-03
hsa04912:GnRH signaling pathway	13	203	98	5085	4,46E-04	1,34E-03
h_biopeptidesPathway:Bioactive Peptide Induced Signaling Pathway	10	124	30	1437	5,62E-04	1,05E-02
h_il3Pathway:IL 3 signaling pathway	7	124	14	1437	5,91E-04	1,01E-02
h_gleevecpathway:Inhibition of Cellular Proliferation by Gleevec	8	124	19	1437	6,00E-04	9,46E-03
hsa04810:Regulation of actin cytoskeleton	20	203	215	5085	7,94E-04	2,32E-03
h_keratinocytePathway:Keratinocyte Differentiation	11	124	38	1437	8,75E-04	1,28E-02
hsa04960:Aldosterone-regulated sodium reabsorption	8	203	41	5085	1,00E-03	2,85E-03

h_il2rbPathway:IL-2 Receptor Beta Chain in T cell Activation	10	124	33	1437	1,21E-03	1,65E-02
h_trkaPathway:Trka Receptor Signaling Pathway	6	124	11	1437	1,29E-03	1,64E-02
h_integrinPathway:Integrin Signaling Pathway	10	124	34	1437	1,53E-03	1,84E-02
h_nfkbPathway:NF-kB Signaling Pathway	8	124	22	1437	1,63E-03	1,85E-02
h_tffPathway:Trefoil Factors Initiate Mucosal Healing	8	124	22	1437	1,63E-03	1,85E-02
hsa05130:Pathogenic Escherichia coli infection	9	203	57	5085	1,67E-03	4,62E-03
h_tidPathway:Chaperones modulate interferon Signaling Pathway	7	124	17	1437	1,96E-03	2,11E-02
h_erkPathway:Erk1/Erk2 Mapk Signaling pathway	9	124	29	1437	2,12E-03	2,16E-02
h_stressPathway:TNF/Stress Related Signaling	8	124	24	1437	2,86E-03	2,77E-02
hsa04060:Cytokine-cytokine receptor interaction	21	203	262	5085	3,45E-03	9,30E-03
h_il6Pathway:IL 6 signaling pathway	7	124	19	1437	3,73E-03	3,44E-02
h_insulinPathway:Insulin Signaling Pathway	7	124	19	1437	3,73E-03	3,44E-02
h_igf1Pathway:IGF-1 Signaling Pathway	7	124	19	1437	3,73E-03	3,44E-02
h_cd40Pathway:CD40L Signaling Pathway	6	124	14	1437	4,52E-03	3,97E-02
h_rnaPathway:Double Stranded RNA Induced Gene Expression	5	124	9	1437	4,59E-03	3,87E-02
h_pyk2Pathway:Links between Pyk2 and Map Kinases	8	124	26	1437	4,71E-03	3,81E-02
hsa05216:Thyroid cancer	6	203	29	5085	5,13E-03	1,35E-02
hsa04623:Cytosolic DNA-sensing pathway	8	203	55	5085	5,65E-03	1,45E-02
h_At1rPathway:Angiotensin II mediated activation of JNK Pathway via Pyk2 dependent signaling	8	124	27	1437	5,91E-03	4,59E-02
h_mapkPathway:MAPKinase Signaling Pathway	15	124	80	1437	6,09E-03	4,56E-02
h_spryPathway:Sprouty regulation of tyrosine kinase signals	6	124	15	1437	6,32E-03	4,55E-02
hsa04914:Progesterone-mediated oocyte maturation	10	203	86	5085	6,69E-03	1,68E-02
hsa04540:Gap junction	10	203	89	5085	8,35E-03	2,05E-02
hsa04330:Notch signaling pathway	7	203	47	5085	1,01E-02	2,41E-02
hsa04310:Wnt signaling pathway	13	203	151	5085	1,63E-02	3,81E-02
hsa04730:Long-term depression	8	203	69	5085	1,89E-02	4,31E-02

[a]Term : name of the pathway
[b]Count : number of proteins of the sub-network involved in the pathway
[c]List total: number of proteins of the sub-network involved in at least one pathway of the database (KEGG or BIOCARTA)
203 proteins of the sub-network are involved in at least one KEGG pathway and 124 in at least one BIOCARTA pathway.
[d]Background 1: number of background (all proteins of the human interactome) proteins involved in the pathway
[e]Background 2: number of background (all proteins of the human interactome) proteins involved in at least one pathway of the database : 5085 proteins of the background are involved in at least one KEGG pathway and 1437 in at least one BIOCARTA pathway.
[f]Pvalue : p-value of the EASE score
[g]Benjamini : corrected p-value with Benjamini-Hochberg method
