# Supplementary figures and images for: Genetic Predisposition to the Mortality in Septic Shock Patients: From GWAS to the Identification of a Regulatory Variant Modulating the Activity of a CISH Enhancer
Source: Int J Mol Sci. 2021 May 29;22(11):5852. doi: 10.3390/ijms22115852 (PMC8198806; doi:10.3390/ijms22115852)

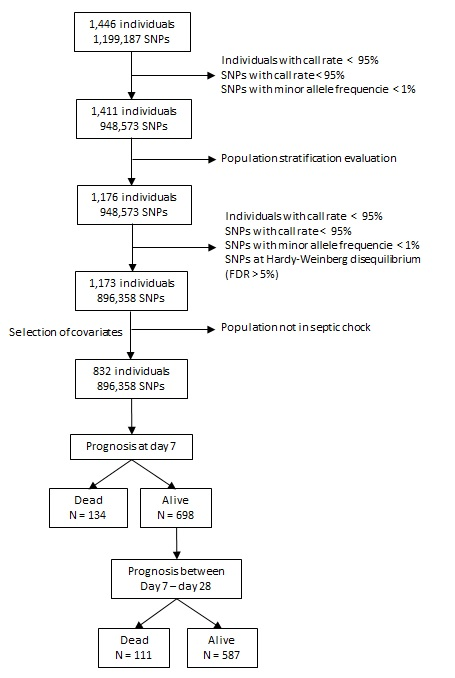

Supplement: Supplementary file 1 [file ijms-22-05852-s001.zip › ijms-1214840 suppl/Supplementary files/Supplementary Figure 1.tiff]
